# Deep-Learning-Derived Facial Electromyogram Signatures of Emotion in Immersive Virtual Reality (bWell): Exploring the Impact of Emotional, Cognitive, and Physical Demands

**DOI:** 10.3390/s26061827

**Published:** 2026-03-13

**Authors:** Zohreh H. Meybodi, Francis Thibault, Budhachandra Khundrakpam, Gino De Luca, Jing Zhang, Joshua A. Granek, Nusrat Choudhury

**Affiliations:** 1 Medical Devices Research Centre, National Research Council Canada, Boucherville, QC J4B 6Y4, Canada; francis.thibault@cnrc-nrc.gc.ca (F.T.); budhachandra.khundrakpam@cnrc-nrc.gc.ca (B.K.); gino.deluca@cnrc-nrc.gc.ca (G.D.L.); 2 Toronto Research Centre, Defence Research & Development Canada, Toronto, ON M3K 2C9, Canada; jing.zhang2@ecn.forces.gc.ca (J.Z.); josh.granek@drdc-rddc.gc.ca (J.A.G.)

**Keywords:** virtual reality (VR), facial electromyography, deep learning (DL), convolutional neural networks (CNN), temporal convolutional networks (TCN), facial expression recognition, emotion recognition, NASA task load index (NASA-TLX), cognitive demand, workload assessment

## Abstract

**Highlights:**

This study investigates the potential of using spatio-temporal deep learning to analyze facial electromyogram (fEMG) signals in immersive virtual reality (VR) environments. By examining the influence of emotional, cognitive, and physical demands, the research aims to capture distinct psychophysiological patterns and link them to nuanced and workload-related affective states in VR settings.

**What are the main findings?**

- A CNN–TCN model trained on physiologically normalized multi-channel fEMG signals classified four calibrated facial expressions (smile, frown, raised eyebrow, neutral) in immersive VR, achieving strong leave-one-participant-out performance (Macro-F1 = 0.88 ± 0.13; ROC-AUC = 0.95 ± 0.06). This was achieved in a small study with only 12 participants, demonstrating the model’s potential and paving the way for further research with larger samples.
- When applied to unlabeled fEMG recordings from previously unseen VR scenes, the trained model generated continuous expression classes, from which static and temporal features showed scene-dependent patterns. These features showed significant associations primarily with perceived physical demand (NASA-TLX), suggesting effective capture of expressions related to physical effort, while associations with cognitive or emotional demand were less pronounced.

**What are the implications of the main findings?**

- End-to-end spatio-temporal modeling of raw fEMG enables privacy-preserving facial expression sensing in immersive VR without handcrafted feature engineering or scene-specific retraining, using a single physiologically normalized model shared across participants. This demonstrates the feasibility of expression monitoring suitable for automated and potentially real-time deployment.
- The convergence between model-derived expression dynamics and NASA-TLX workload ratings showcases the potential for reducing reliance on intermittent self-report measures in future VR applications. By bridging brief calibration-based learning with spontaneous task-elicited behavior, the framework supports continuous, physiologically grounded assessment that can complement, and in some contexts, partially substitute for, explicit questionnaires in training, performance evaluation, and user-experience research.

**Abstract:**

Emotional and workload-related states unfold dynamically during immersive virtual reality (VR) experiences, yet reliable physiological modeling in such environments remains challenging. We investigated whether multi-channel facial electromyography (fEMG), combined with spatio-temporal deep learning, can (i) accurately classify calibrated facial expressions across participants and (ii) transfer to spontaneous, task-elicited behavior in immersive VR. Twelve adults completed a calibration phase involving four intentional expressions (smile, frown, raised eyebrow, neutral), followed by VR scenes designed to elicit emotional, cognitive, physical, and dual task demands. After participant-level physiological normalization, a single shared Convolutional Neural Network–Temporal Convolutional Network (CNN–TCN) model was trained and evaluated using leave-one-participant-out (LOPO) validation. The model achieved strong cross-participant performance (Macro-F1 = 0.88 ± 0.13; ROC-AUC = 0.95 ± 0.06). When applied to unlabeled spontaneous VR task-elicited fEMG recordings, the trained model generated continuous expression classes. Derived static and temporal expression features showed scene-dependent modulation and False Discovery Rate (FDR)-surviving associations, primarily with perceived physical demand (NASA-TLX). The observed muscle activation patterns were physiologically plausible and aligned with Facial Action Coding System (FACS)-based interpretations of underlying muscle activity. These findings demonstrate that end-to-end spatio-temporal modeling of raw fEMG enables facial expression sensing in immersive VR using a single shared model following physiological normalization. The proposed framework bridges calibrated expression learning and spontaneous task-elicited behavior, supporting privacy-preserving, continuous and physiologically grounded monitoring in human-centered VR applications.

## 1. Introduction

Emotions shape our daily lives, influencing our behavior, perception and cognitive processes. As such, understanding and recognizing emotional responses is vital for advancing human research. Emotions are complex physiological and psychological responses to internal or external stimuli, producing coordinated changes in bodily states, cognitive processes, and expressive behavior [1]. Facial expressions represent one of the most immediate and informative channels through which these emotional responses manifest [2]. These systems are traditionally grounded in the Facial Action Coding System (FACS) [3], which links observable facial movements to underlying emotional processes. Modern implementations typically rely on image- [4,5,6] or video-based algorithms [7,8,9], to detect the facial action units (AU) associated with basic emotions such as happiness, fear, anger, and surprise. Although these approaches have achieved substantial progress, they present notable limitations, including privacy concerns related to video capture, sensitivity to lighting and occlusion, and reduced performance when facial expressions are subtle or partially obstructed [10,11,12,13].

To overcome these limitations, there is growing interest in physiological approaches to emotion detection. In particular, facial electromyography (fEMG) offers a way to measure the underlying muscle activation associated with expressive behavior [14,15,16]. Because fEMG captures neuromuscular signals directly, it can detect affective responses even when visible facial features are partially or fully obscured and offers a privacy-preserving alternative to camera-based methods [17]. Prior works [18,19,20,21,22] have shown that fEMG signals exhibit high signal-to-noise ratios and correlate strongly with both spontaneous and intentional affective expressions, highlighting their potential for continuous emotion monitoring.

Immersive virtual reality (VR) is increasingly used to elicit an ecologically valid range of emotional responses [23,24,25,26,27]. Through standardized and interactive scenarios, VR can evoke a broad range of context-dependent affective and workload-related states, including stress, cognitive effort, and temporal pressure, while enabling repeatable experimental conditions [28,29,30,31]. However, the occlusion of facial features by VR headsets limits the reliability of vision-based facial expression analysis, creating a need for alternative sensing modalities that remain robust during immersive interaction [32].

To capitalize on VR’s advantages and address the limitations of its use with vision-based emotion detection, the use of fEMG sensors with VR is crucial, enabling unobstructed measurement of facial muscle activity during immersive experiences. fEMG has been shown to reliably reflect affective valence and basic emotional responses by providing a direct measure of muscle activity associated with expressive behavior, with studies demonstrating its robustness to subtle or masked expressions [33,34]. Early studies showed that activity in muscles such as the zygomaticus major reliably reflects affective valence and basic emotional responses [35]. Later work expanded to multi-channel fEMG systems, extracting handcrafted time- and frequency-domain features for distinguishing discrete emotions [36]. Despite its advantages, fEMG-based emotion detection faces several challenges. There is no standardized protocol for acquisition or analysis, resulting in variability across studies. Most existing work focuses on basic, discrete emotions and does not capture the nuanced affective states present in naturalistic tasks [37]. fEMG signals also exhibit substantial inter-individual variability in muscle anatomy and activation patterns, necessitating personalized modeling approaches for reliable decoding. Contextual factors—such as task demands, environment, and cognitive load—further modulate facial muscle activity, complicating interpretation [33]. Moreover, conventional modeling and analysis pipelines often rely on traditional feature-engineering approaches that struggle to capture how expressions evolve over time, especially during cognitively or emotionally dynamic VR tasks [38].

These challenges are amplified in immersive VR environments, where emotional, cognitive, and physical demands evolve continuously, producing complex spatio-temporal activation patterns across facial muscles [39,40,41]. Effective modeling of VR-based fEMG therefore requires analytical frameworks capable of capturing temporal dependencies, multi-muscle coordination, and individual variability. Spatio-temporal deep learning approaches offer a principled solution by enabling automatic extraction of meaningful patterns from high-dimensional physiological data while preserving temporal structure and inter-channel relationships.

Building on this framework, the National Research Council Canada (NRC) has previously developed bWell, an interactive and immersive VR platform designed as a broadly applicable toolkit delivering multisensory tasks, targeting general aspects of cognition and everyday functioning [42,43,44]. This platform is currently being utilized within a larger program of research aimed at integrating VR with multimodal physiological data for systematic and individualized understanding of stress response. The comprehensive methodology for the development of this platform for this target application, including design, implementation and exercises, are described in detail in our previous publication [45]. In a preliminary pilot study, multimodal data—including heart rate, fEMG, perceived stress, and demographic information—were used to develop supervised models predicting subjective task load during VR-based stress exposure [46]. These findings identified facial expression-derived fEMG features among the strongest predictors of perceived workload, motivating the present study’s focused analysis of fEMG signals and exploratory development of an end-to-end processing and modeling pipeline grounded in FACS-informed interpretation.

In this work, we present a unified framework integrating immersive VR, multi-channel fEMG, and a Convolutional Neural Network–Temporal Convolutional Network (CNN–TCN) spatio-temporal deep learning architecture for expression modeling and workload-related analysis. Within this architecture, the CNN component captures spatial patterns and inter-channel relationships in physiologically normalized fEMG signals, while the TCN models temporal dependencies and dynamic evolution of muscle activity over time. The approach leverages physiologically normalized signals and a shared model trained across participants to classify calibrated facial expressions from brief standardization trials. We then extend this framework to spontaneous, VR task-elicited fEMG recordings to infer continuous expression dynamics and extract static and temporal features characterizing expressive behavior across experimental scenes. Finally, we evaluate whether these fEMG-derived dynamics exhibit scene-dependent patterns and statistically robust associations with subjective workload ratings, while examining their consistency with FACS-aligned facial action patterns. The goal of this approach is not to generalize to a larger population but rather create a model calibrated with data from a specific cohort, allowing for precise inference within that same group. We hypothesize that this approach would be similar to a data encoding process, minimizing the necessity for a large cohort to develop a potentially broadly applicable model for predicting outcomes in new populations. Together, these contributions establish a scalable, privacy-preserving, and physiologically grounded foundation for automated expression monitoring and workload-related assessment in immersive VR environments.

## 2. Materials and Methods

In this study, participants are immersed in diverse virtual scenarios specifically designed to elicit a spectrum of emotions. These include basic emotion labels such as frustration and calm, as well as more nuanced labels that reflect the intersection of emotional and cognitive processes, such as determination, interest, anticipation, and task load. To effectively capture these emotional responses, this study utilizes both self-reports and continuous physiological measures. This data collection supports the development of a DL model.

A total of 12 participants were recruited for this study (6 females, 6 males). Female participants ranged in age from 29 to 45 years (*M* = 38.7, *SD* = 7.5), and male participants ranged from 25 to 56 years (*M* = 42.5, *SD* = 10.1). Participants were healthy volunteers with no known neurological disorder, head trauma, or other unstable medical conditions which may adversely impact cognitive functioning or presence of any physical mobility issues that limit use of VR. Personal characteristics data were obtained including age, sex, handedness, gaming activity and VR experience. The participants were recruited based on their motivation to participate in the study. Before the experiment, all participants received a detailed explanation of the experimental protocols. Subsequently, they approved and signed a written consent form. Ethics approval for the study was obtained from the Research Ethics Board (REB) of the National Research Council Canada (NRC 2020 and NRC 2023-46) and from Defence Research and Development Canada Human Research Ethics Committee (HREC 2022-032).

**Figure 1:**
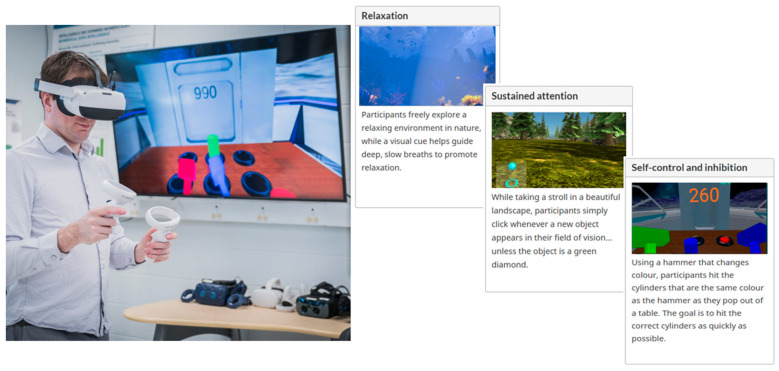
bWell platform laboratory set-up with user immersed in a virtual reality environment. It is designed to be hardware agnostic, allowing compatibility with a wide range of commercially available VR headsets. The platform is transdiagnostic, focusing on cognitive domains relevant to a broad range of physical, mental, and behavioral conditions. Featured: three of the exercises selected from the bWell battery for the current study, targeting relaxation for baseline/recovery from task demand, sustained attention (continuous performance task) and self-control and inhibition (Go/No-Go task). In the self-control and inhibition task, colored cylinders represent selectable targets; participants must hit the cylinders matching the hammer color.

bWell has been designed with a suite of customizable tasks, allowing users to select tasks tailored to the unique needs of various fields or target populations. The platform has been adapted for specific applications, including multimodal assessment of stress response [45,46], cognitive remediation for depression [47,48], exploring memory across aging [49], and integration with electroencephalography (EEG) [50,51]. The tasks used in the current study are briefly summarized in Figure 1 and detailed as follows:

**Tent**—nature scene to collect data on user while immersed in VR but not required to perform a task. Used for resting/baseline state and recovery after a task.

**City**—passive viewing of a busy city with noise pollution—roads with ongoing traffic, including honking cars and sirens. Used to elicit an emotional response.

**Mole**—an exercise targeting response inhibition and cognitive control in which the user has a hammer in each hand and must hit cylinders that pop up in front of them (only when the cylinder and hammer are of the same color).

**Stroll**—an exercise administered as a purely physical demand (Stroll) and/or a dual task with both physical and cognitive demand (Stroll + CPT). To exert physical demand, the user must physically displace themselves to advance in the scene. The user was asked to jog, keeping their heart rate at a moderate level of exertion (the predefined threshold was displayed as a gauge based on individual heart rate). As a dual task, the exercise includes a secondary task targeting sustained attention with a continuous performance test (CPT) in which the user must press a button for each new shape that appears, except when it is a green diamond.

**Figure 2:**
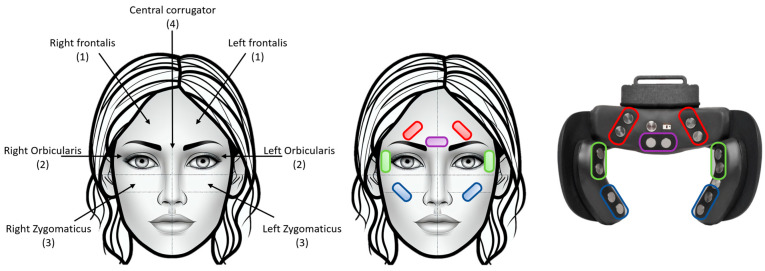
emteqPRO multi-sensor face mask, including seven-channel EMG. Mapping between the EMG sensors on the device and facial muscles are shown: (1) frontalis (red), (2) orbicularis (green), (3) zygomaticus (blue) and (4) corrugator (purple), adapted from [53].

fEMG was collected using a commercially available system, the emteqPRO by Emteq Labs (Brighton, UK), which consists of a sensor mask insert for VR headsets [52]. The mask is equipped with seven dry facial EMG electrodes (Figure 2) to measure facial muscle activity detection, positioned at the (1) frontalis (eyebrow), (2) orbicularis (eye), (3) zygomaticus (mouth and cheek) and (4) corrugator (forehead). The signals are sampled at a frequency of 1000 Hz. In contrast to traditional camera-based systems, this technology can capture subtle facial movements in order to provide insights into a user’s affective state and enable application in virtual reality. In this study, the emteqPRO system was integrated into the HTC Vive Pro Eye VR head-mounted display (HTC Corporation, New Taipei City, Taiwan).

The emteqPRO focuses on key muscles identified in the FACS, which provides a comprehensive framework for identifying facial muscles involved in various expressions [3], summarized in Table 1. Specifically, it targets muscles such as the zygomaticus major, which is primarily responsible for pulling the lip corners upward, contributing to smiling. It also monitors the corrugator supercilii muscle that is involved in frowning, as it draws the eyebrows downward and together, creating vertical lines between the brows. The frontalis muscle, responsible for elevating the eyebrows, is targeted to capture expressions of surprise or attentiveness. Additionally, the orbicularis oculi muscle, which encircles the eyes, is key for facilitating eye closure, often associated with genuine smiles (Duchenne smiles) [54]. By capturing the activity of these muscles, the emteqPRO can effectively monitor a subset of emotions outlined in the FACS, such as happiness, sadness and surprise.

**Figure 3:**
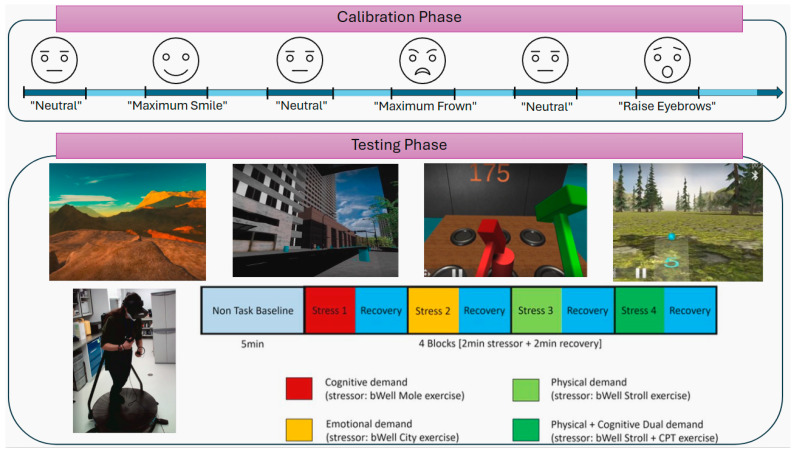
Overview of the experimental protocol, illustrating the calibration phase—during which participants performed intentional facial expressions—and the testing phase, consisting of first a baseline (1st image), followed by a pseudo-randomized sequence of stressor scenes designed to elicit emotional (2nd image), cognitive (3rd image), physical (bottom image), and combined (dual) demands (4th and bottom image), each followed by a recovery scene.

The study was designed to systematically elicit specific emotional, physical, cognitive, and dual task demands by engaging participants in a series of VR environments, as illustrated in Figure 3. In alignment with recent related work [55], the study protocol began with an initial calibration phase (included in the emteqPRO software, Emteq SDK V2) where participants were asked to intentionally make specific facial expressions. This phase aimed to generate a high-quality, precisely annotated dataset of facial expressions suitable for training a predictive model. The facial expressions used in this study were limited to four expressions (smile, frown, raised eyebrow, and neutral), dictated by the standardized emteqPRO calibration protocol [52,53], and employed as expression-level markers rather than direct representations of complex emotional states. Further, participant-specific calibration enabled robust normalization across individuals. During the calibration, participants followed onscreen instructions prompting them to hold distinct expressions—such as maximum smile, maximum frown, and eyebrow raise (surprise)—for a few seconds each, while maintaining a neutral expression in between. During the experimental testing phase, participants were exposed to a sequence of VR environments designed to elicit distinct physiological and psychological responses. The session started with a neutral nature scene where participants were instructed to remain stationary, standing with their eyes open. This resting condition served to establish a 5 min baseline measurement of heart rate variability (HRV), consistent with established guidelines for HRV data collection and analysis [56]. Following this baseline period, participants entered the main testing phase, which comprised a succession of interactive VR environments. Four active stressor scenes, each lasting two minutes, are designed to elicit specific demands: one primarily a cognitive load, another emotional stress, a third focusing on physical exertion, and a fourth combining both physical and cognitive demands, representing a dual task that mirrors real-world challenges. These environments were respectively the Mole, City, Stroll, and Stroll CPT exercises. To facilitate physiological and psychological recovery between active stress exposures, each stressor scene was followed by a two-minute presentation of a passive and tranquil VR environment depicting a natural setting devoid of targeted stimuli. This recovery condition was the Tent nature scene. Participants remained standing for all tasks except during the physical and dual cognitive-physical conditions, where they were instructed to run on a VR treadmill, as illustrated in Figure 3. All participants experienced each of the active stressor environments; however, the order of presentation was pseudo-randomized across individuals to control potential sequence and carryover effects. This structured approach enabled the capture of both psychophysiological signals and facial expressions in a dynamic yet regulated setting, facilitating a comprehensive analysis of how various stressors uniquely affect emotional responses.

**Figure 4:**
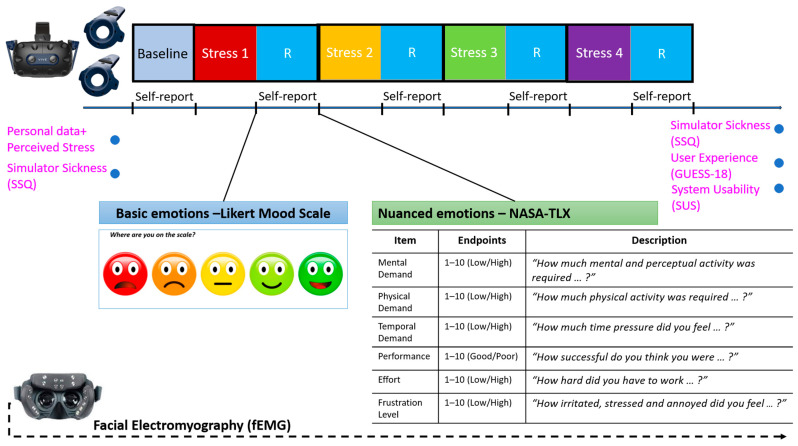
Distribution of participants’ self-assessed emotional responses and data collection overview. Participants completed demographic surveys and three questionnaires: SSQ, GUESS-18 and SUS. Simulator sickness was evaluated before and after VR exposure. Participants also conducted ratings while in VR, and after each stressor scene, using a 5-point emoji Likert scale and workload with the NASA-Task Load Index (NASA-TLX). Continuous fEMG data were collected during testing. SSQ = Simulator Sickness Questionnaire, GUESS-18 = Game User Experience Satisfaction Scale and SUS = System Usability Scale.

Participant demographic data and three standardized self-report questionnaires were administered to explore the effects of potential covariates and confounding factors. These were the Simulator Sickness Questionnaire (SSQ) [57], the short version of the Game User Experience Satisfaction Scale (GUESS-18) [58] and the System Usability Scale (SUS) [59]. Simulator sickness was evaluated at baseline and after VR exposure. Facial expressions data were continuously collected throughout the whole testing phase using fEMG. Participants provided subjective mood (5-point emoji-based Likert scale) and workload ratings for each stressor task during the corresponding recovery periods. The workload was captured using the NASA Task Load Index (NASA-TLX) [60], a widely used tool for assessing perceived workload across various dimensions. While it is not specifically designed to capture emotions directly, it can provide insights into the emotional states of participants by measuring the mental, physical, and temporal demands of a task, as well as perceived performance, effort, and frustration levels. Figure 4 illustrates the distribution of participants’ self-assessed emotional responses, with the two types of self-report measures. After completing the session, participants reviewed a screen recording of their virtual task performance and provided qualitative feedback. They also completed the series of questionnaires to assess perceptions related to user experience and satisfaction, and system usability.

All data collected in this study were ingested and processed using a Python-based framework (v3.10.12) using PyCharm (v2025.3.3), developed by the NRC. This framework streamlines the aggregation of time-resolved, multimodal data and facilitates efficient feature extraction over custom-defined time intervals, thereby enabling subsequent statistical and deep learning analyses. The data processing pipeline used to construct the customized deep learning (DL) model comprised three main stages: (1) data tagging, (2) normalization, and (3) sliding-window segmentation.

**Tagging:** The emteqPRO provides filtered EMG data, free of noise and irrelevant frequency components providing a clean representation of muscle activity, and from which EMG amplitude is derived. The amplitude correlates with the degree of muscle activation; stronger muscle activation results in higher voltage amplitude. This measure provides direct insight into the power of fEMG activation at a given time for analyzing facial expressions and emotional responses. The data was tagged according to the experimental context:

Calibration data were labeled with the intentional facial expression elicited during each trial (smile, frown, eyebrow-raise, neutral).

Experimental, testing phase, recordings were tagged with scene-level metadata describing the emotional, cognitive, physical, and dual-demand VR stressors.

This tagging ensured that each segment of signal data was linked to the appropriate expression label or task condition for downstream modeling and statistical analysis.

**Normalization:** To account for participant-specific physiology and reduce inter-individual variability, fEMG must be normalized [61]. Based on recommendations from earlier studies [12], we applied two successive normalization procedures to ensure that the data is comparable across participants: Neutral correction and Maximum Voluntary Isometric Contraction (MVIC). Neutral correction specifically targets baseline differences in resting tone across participants and ensures that the signals reflect task-related activation rather than individual baseline drift. It is significant because it standardizes the starting point for each participant’s data, allowing for more accurate comparisons. MVIC normalization scales the muscle activation relative to individual maximum capacity, thereby reducing inter-individual variability in signal amplitude associated with differences in muscle strength and recruitment. This step is vital for ensuring that the fEMG amplitudes are comparable across individuals, as it accounts for physiological differences in muscle activation capabilities. Details on these two normalization procedures, including the specific steps, illustrations, and their effects, are provided as (Appendix A.1).

**Sliding-Window Segmentation:** Sliding-window segmentation was used as a data-augmentation method by dividing each participant’s continuous normalized fEMG signals into fixed-length samples for model training, thereby increasing the number of training instances per expression. Each signal was labeled according to the elicited facial expression. Signals were divided into overlapping windows of length *w* with a stride of *s*; only complete windows containing the full number of samples were retained. In this work, the window length was determined empirically through pilot testing to balance signal stability and temporal resolution, and a zero-overlap stride was used to minimize redundancy and reduce overfitting from highly correlated samples.

**Figure 5:**
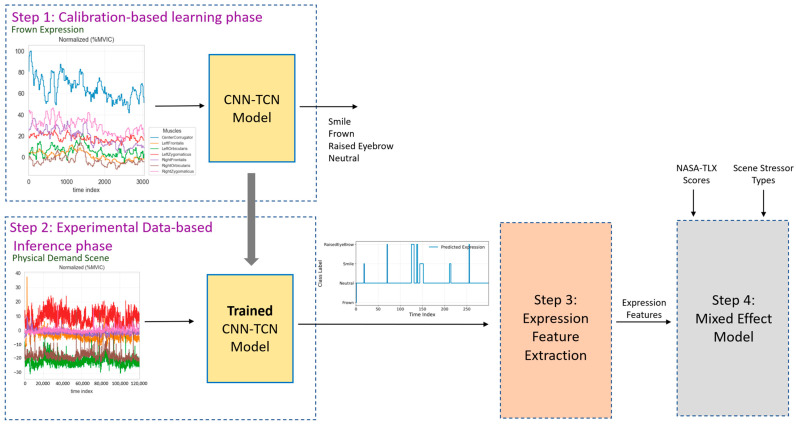
Diagram of the fEMG expression modeling pipeline, which includes (1) training the model on labeled calibration data, (2) applying the trained model on unlabeled experimental data, (3) extracting features from the predicted facial expressions, and (4) mixed-effects statistical analysis to relate predicted facial expression dynamics from fEMG signals to self-reported workload across various VR conditions (emotional, cognitive, physical and dual demands). Colored lines in the signal plots correspond to the seven fEMG muscle channels: blue = center corrugator, orange = left frontalis, green = left orbicularis, red = left zygomaticus, purple = right frontalis, brown = right orbicularis, and pink = right zygomaticus.

Figure 5 presents an overview of the expression modeling pipeline, which integrates calibration-based normalization, model training, expression-feature extraction, and mixed-effects statistical analysis. In the calibration phase (Step 1 in Figure 5), fEMG signals are normalized using participant-specific MVIC values computed from calibration recordings. All preprocessing and MVIC normalization were performed independently for each participant prior to cross-validation. Because MVIC normalization relies solely on each participant’s own calibration data and does not involve shared statistics or learnable parameters, it does not introduce data leakage. After normalization, calibration data from all participants are pooled to train a single shared CNN–TCN model that learns population-level patterns of facial muscle activation associated with different expressions. Model performance was evaluated using leave-one-participant-out (LOPO) cross-validation, ensuring that calibration data from the held-out participant were excluded from training in each fold. During the inference phase (Step 2), the finalized model, trained on the full pooled calibration dataset after cross-validation, is applied to normalized, unlabeled experimental fEMG recordings collected during the VR stressor scenes to generate continuous time-series of predicted facial expressions. These expression sequences are then transformed into a set of statistical expression-based features for each participant and scene (Step 3). Finally, these features are combined with NASA-TLX self-reported workload scores in a mixed-effects modeling framework to quantify how predicted expression dynamics relate to subjective workload across the emotional, cognitive, physical, and dual-demand VR scenes (Step 4). In the following, each component of the pipeline is described in detail.

**Figure 6:**
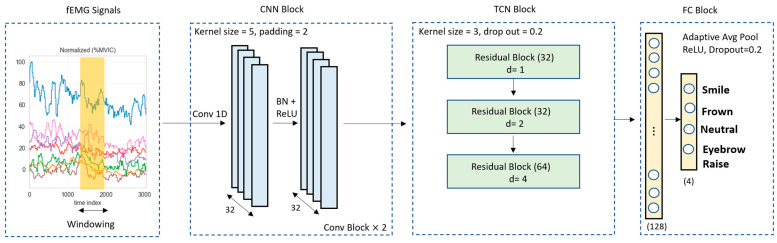
CNN-TCN model architecture used for facial expression classification. Normalized multi-channel fEMG data is input to the model, where spatio-temporal features are successively extracted in each of the three blocks: (1) CNN, (2) TCN and (3) FC. fEMG = facial electromyography, CCN = Convolutional Neural Network, TCN = Temporal Convolutional Network and FC = Fully connected.

Step 1 involves training the model using the calibration phase fEMG signals captured from seven channels, including the left and right orbicularis, left and right zygomaticus, left and right frontalis, and a single corrugator channel. During this phase, participants are explicitly instructed to perform a specific facial expression, including smiling, maintaining a neutral face, frowning, and raising eyebrows, used to label the data. The model is trained on the calibration dataset to learn the temporal and spatial representations of the four intentional facial expressions. By using the data obtained from the calibration phase to train the model, the model’s ability to interpret and classify facial expressions based on muscle activity is significantly enhanced. For training, we employed the LOPO strategy, where each participant’s data is used as a test set once, while data from all remaining participants are used for model development. Within each fold, the training portion was further split into training and validation subsets to enable hyperparameter tuning and early stopping. This process is repeated for each participant, ensuring that the model is evaluated on all participants’ data. Following this generalizability analysis, the model was retrained using the full calibration dataset, and the resulting model was used for all subsequent analyses in the second phase of the study. As illustrated in Figure 6, the CNN-TCN model consists of three main components: CNN Block, TCN Block, and Fully Connected (FC) Block, with their configurations and hyperparameters provided as (Appendix A.2).

In Step 2, the inference phase, the CNN–TCN model trained on the normalized calibration fEMG signals is applied to the full set of unlabeled normalized experimental fEMG recordings collected during the VR stressor sessions. Each participant’s continuous fEMG signals are segmented into windows using the same windowing procedure as in training, and the trained model produces a class prediction for each window corresponding to the most likely facial expression. These class predictions are then assembled into a continuous time-series sequence of expressions for each VR scene. This inference process allows us to estimate spontaneous, natural facial expressions as they unfolded throughout the emotional, cognitive, physical, and dual-demand VR conditions, without requiring any manual labels during the experiment. The resulting expression time-series served as the input for the subsequent feature extraction stage, enabling a detailed analysis of expression dynamics across different stressor conditions. It is worth mentioning that although participant-specific calibration recordings are used, they serve exclusively for MVIC-based normalization of facial EMG signals rather than participant-specific model training. After normalization, a single shared CNN–TCN model is trained on pooled data from all participants. The model therefore operates as a scene-agnostic, cross-validated framework within the current sample, but not as a calibration-free system.

In Step 3, after generating continuous time-series of predicted expressions in the inference phase, the next step involves deriving statistical features that summarize meaningful aspects of expression behavior. Depending on the application, the pipeline could end at expression prediction, for real-time expression recognition or monitoring. However, in this study, we aim to go beyond expression classification and investigate whether facial expression dynamics could reveal more nuanced patterns related to cognitive, emotional, physical, and mental workload. To do so, a comprehensive set of expression-based features is computed from the predicted facial expression time-series. Feature extraction is performed separately for each participant and each VR scene. In total, the continuous fEMG recordings were segmented into fixed temporal windows for model training, yielding a substantial number of spatio-temporal samples per participant. This windowing strategy increased temporal data density and supported stable optimization of the CNN–TCN model within the participant-independent evaluation framework. This extensive data helps mitigate the limitations typically associated with a small sample size and enhances statistical power. The features captured multiple aspects of temporal expression behavior and are grouped into four conceptual categories. The comprehensive list of features and details on the categories is provided as (Appendix A.3). The resulting expression features served as inputs to the mixed-effects modeling stage, where their relationship to self-reported workload measured by NASA-TLX is examined.

In Step 4, to examine whether facial expression dynamics reflect the demands elicited by each VR stressor condition, a regression analysis is performed using the extracted expression features together with self-reported workload scores from the NASA-TLX. Mixed-effects model is chosen because it accounts for repeated measures within participants as a random effect and enables estimation of scene-specific effects while controlling for individual variability. For each expression feature, its relationship with the NASA-TLX subscales is modeled to determine whether specific aspects of facial expression behavior correspond to participants’ subjective experiences of mental, emotional, physical, or temporal workload. Since there are 8 NASA-TLX subscale scores, we performed corrections for multiple comparisons using False Discovery Rate (FDR) [62]. This step aims to determine whether the predicted expression patterns meaningfully reflect the stressor by identifying expression features most strongly associated with each scene demand. In other words, given a VR scene designed to elicit cognitive effort, emotional frustration, physical exertion, or combined dual demand, we test whether the expression features respond in ways that aligned with the intended manipulation and are consistent with established FACS interpretations. Scene-specific associations (e.g., increased frown-related dynamics during cognitive tasks or increased lower-face activation during physical tasks) indicate that the model captures physiologically plausible responses tied to the nature of the workload.

## 3. Results

Before proceeding with the model-based analyses, we conducted a series of verification analyses to ensure that (i) the VR scenes successfully elicited the intended emotional and workload states and (ii) the recorded fEMG signals exhibited physiologically coherent and reliable activation patterns. These analyses included statistical evaluation of self-reported emotional ratings, NASA-TLX workload distributions across scenes, bilateral fEMG reliability testing, validation of expression-specific activation signatures during calibration, and examination of spontaneous scene-dependent fEMG modulation during the experimental phase. To maintain focus on the primary modeling outcomes in the main Results section, detailed statistical procedures, figures, and tables supporting these validation analyses are provided as (Appendix A.4, Appendix A.5 and Appendix A.6). Briefly, the self-reported mood ratings via the emoji-based Likert scale showed significant variability across scenes, with a Friedman test revealing a significant main effect of scene, χ^2^(4) = 10.93, *p* = 0.027, and a Kendall’s W = 0.23 indicating a small-to-moderate effect size. Post hoc comparisons confirmed significant differentiation between the emotional and cognitive demanding scenes (p_adj_ = 0.011), validating the sensitivity of the scale to scene-induced emotional experiences. NASA–TLX subscale scores were examined to evaluate whether the four VR scenes elicited distinct workload profiles. Friedman tests indicated significant scene effects for mental, physical, temporal demand, performance, and effort, all with *p*-values < 0.05, but not frustration, confirming that most of the VR exercises elicited distinct workload profiles consistent with their intended task demands. Post hoc Wilcoxon tests showed that mental demand was higher in the dual-task condition than in the purely physical condition, and that emotional demand scene was rated as less physically demanding than both cognitive and dual tasks, aligning with the intended task manipulations, validating the experimental manipulations. The continuous sensor-based measures were also verified. Spontaneous fEMG dynamics revealed distinct scene-specific muscle activation patterns, with significant increases in frontalis activity during the emotionally demanding scene (*p*(FDR) = 0.05), and pronounced zygomaticus and orbicularis activation during physical (respectively *p*(FDR) = 0.05 and 0.04) and dual-task (respectively *p*(FDR) = 0.006 and 0.01) conditions, showing moderate to large effects. Empirically, muscle activations were not continuously sustained but instead appeared as transient peaks that rose and returned to baseline over time. This behavior highlights the inherently dynamic nature of facial muscle responses and underscores the need for temporal modeling approaches such as CNN–TCN.

### 3.1. Demographic and Questionnaire Data

The study featured a balanced gender distribution, with an equal number of female and male participants (6 each), which helps mitigate gender-related biases. However, the average age for male participants was slightly higher (M = 42.5) compared to female participants (M = 38.7). Age variations within this small sample could introduce variability in responses, potentially influenced by factors such as familiarity with technology or susceptibility to simulator sickness.

Participants reported limited routine gaming activity, with an average of 4.0 ± 7.6 h per week (range = 0–20). The most common level of VR experience was “Fair” (“I know of the types of VR—immersive, non-immersive, mixed reality, etc.”), reported by 5 of 12 participants (41.7%), followed by “Very good” (“I own a virtual reality headset”) and “Average” (“I’ve tried VR”), each reported by 3 participants (25%). Most participants used VR less than once a month (7 of 12; 58.3%), and the majority (11 of 12; 91.7%) described themselves as extremely comfortable with technology. Despite the variability in VR experience, it is noteworthy that none of the participants reported having “No experience with VR”. Coupled with the high level of technological comfort reported by the majority, we do not anticipate a significant impact on emotional responses solely due to variations in VR experience.

The SSQ assessed symptoms of cybersickness across three primary categories: nausea, oculomotor disturbances, and disorientation. Severity was determined by total SSQ scores: 0–5 (negligible), 5–10 (mild), 10–15 (moderate), and 15+ (severe) [63]. The mean post-session SSQ score was 5.91 ±4.4, with most participants reporting negligible to mild symptoms, as scores fell within the range of 1 to 8. Two participants demonstrated significant symptoms of post-session, with scores of 11 and 14, but they also reported symptoms of pre-session, suggesting that these were not solely due to VR exposure. Overall, cybersickness did not appear to be a significant confounding factor in the study.

The SUS was used to assess whether there were any usability issues, such as difficulties navigating the interface, that might influence participant performance or emotional responses. The SUS yielded an overall mean score of 73.2 ±18.8, indicating above average usability, with scores above 68 generally reflective of good usability. This suggests that the observed effects are unlikely to be due to usability challenges, but rather primarily attributable to the experimental manipulations.

The GUESS-18 was used to examine how different aspects of the gaming might affect participant engagement or emotional responses. Excluding “social connectivity” due to the lack of participant interaction during VR exercises, the maximum score was adjusted to 56. Participants reported an overall positive experience, with scores ranging from 34 to 46, and a mean score of 38.5 ± 4.4. Good satisfaction across GUESS-18 dimensions suggests that observed effects are more likely due to experimental manipulations rather than dissatisfaction with the experience.

### 3.2. Calibration-Based Learning Phase Performance

**Figure 7:**
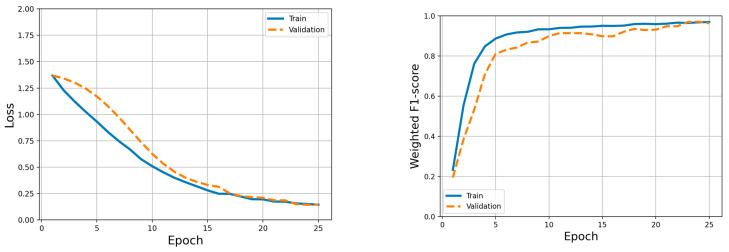
Training dynamics (loss and weighted F1-score) of the CNN–TCN facial expression classifier. On the left shows the model loss over successive epochs, showcasing the convergence behavior during training. On the right presents the weighted F1-score, highlighting the model’s performance improvements.

Overall, the model demonstrated strong performance across training, validation, and held-out test participants. It was seen that a large proportion of expression segments were neutral; as such, model performance was quantified using both weighted and macro-averaged evaluation metrics to ensure that minority expression classes contributed proportionally to the overall assessment. The average classification metrics across all LOPO folds are summarized in Table 2, including accuracy, macro/weighted F1-scores, macro/weighted precision and recall, and Receiver Operating Characteristic Area-Under-the-Curve (ROC-AUC). Test performance remained robust (accuracy ≈ 0.93 ± 0.07; macro-F1 ≈ 0.88 ± 0.13), indicating effective generalization to unseen participants. Training dynamics are illustrated in Figure 7, which shows the evolution of the training and validation loss (left panel) and the weighted F1-score (right panel) over epochs. The rapid decrease in loss and corresponding increase in F1-score during the first epochs, followed by stabilization, confirms effective optimization and the absence of overfitting. These results indicate that the CNN–TCN architecture is capable of reliably extracting participant-specific facial expression representations from multichannel fEMG signals.

**Figure 8:**
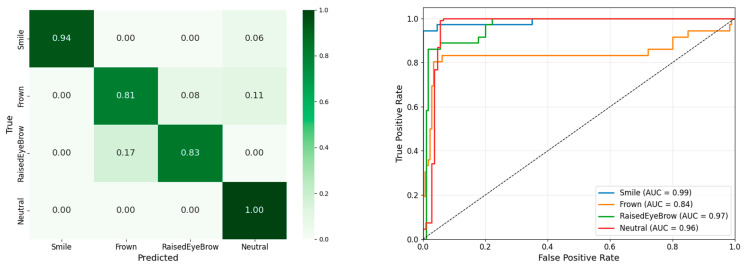
Evaluation results of the LOPO test. On the left displays the confusion matrix, illustrating the classifier’s accuracy across different facial expression categories. On the right presents the ROC curves for each class, highlighting the model’s discriminative ability.

As shown in Figure 8 (left), the LOPO evaluation on the test dataset revealed that the model achieved high classification reliability across all four expressions (smile = 0.94, frown = 0.81, raised eyebrow = 0.83, neutral = 1.00). smiles and neutral faces were identified with very high sensitivity, with only a small fraction of smile trials (6%) being confused with neutral and no other cross-confusions. Raised eyebrow expressions were also classified robustly, with the main source of error being confusion with frown (17%), which is consistent with the partially overlapping activation patterns of these expressions in the corrugator and frontalis channels. Frown showed the lowest recall and was misclassified as neutral (11%) or raised eyebrow (8%), but it nonetheless remained clearly separable from the other categories, indicating that the model still captured the characteristic brow-lowering activation pattern despite inter-individual variability. The ROC curves (Figure 8, right panel) further highlight the classifier’s discriminative strength. All four expressions achieved AUC values above 0.84, and the curves for smile, eyebrow raised, and neutral approached ceiling performance. As shown in the right panel, the consistently high true-positive rates across a wide range of false-positive thresholds indicate robust separability of the underlying fEMG patterns.

After LOPO cross-validation, the CNN–TCN classifier was retrained on the full calibration dataset to produce the final model used for inference. This model demonstrated excellent performance across all four facial expressions, with weighted precision, recall, and F1-scores all exceeding 0.98. Per-class metrics were likewise high: smile (1.00/0.97/0.98), raised eyebrow (1.00/1.00/1.00), neutral (0.96/1.00/0.98), and frown (1.00/0.89/0.94) for precision/recall/F1-score, respectively. Most misclassifications reflected mild confusion with the neutral class, with 3% of smile trials and 11% of frown trials predicted as neutral, whereas raised eyebrow and neutral expressions were never confused with other categories. The overall accuracy of 0.98 further confirms the stability and generalizability of the model. These results indicate that the CNN–TCN architecture learned highly discriminative, physiologically meaningful representations of facial muscle activation, providing a reliable foundation for subsequent extraction of expression features during the VR experimental phase and their analysis via mixed-effects modeling.

### 3.3. Inference-Phase Expression Patterns Across Experimental Scenes

**Figure 9:**
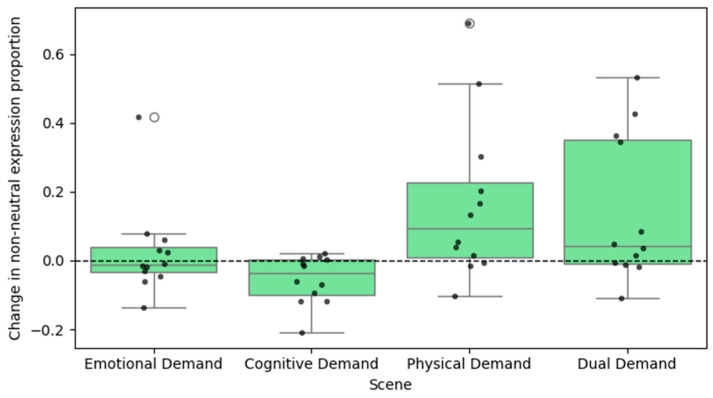
Baseline-corrected non-neutral expressiveness across demand scenes. Expressiveness was minimal during the emotional and cognitive demand scenes but increased significantly during physical and dual demand scenes, with the greatest variability in the dual demand condition. These trends reflect typical suppression under cognitive load and enhanced expressiveness in more physically and multimodally demanding scenarios. Black dots represent individual participant observations, while unfilled circles indicate outliers beyond the whisker range of the boxplot.

Since the experimental recordings did not contain ground-truth expression labels, inference-phase performance was assessed by characterizing scene-level expression patterns, evaluating physiological plausibility, and examining consistency with native Emteq expression indices. The model’s output, a continuous time-series of predicted expression classes, was summarized at the participant level by computing the proportion of non-neutral expressions in each scene and quantifying each participant’s change relative to their own baseline. As shown in Figure 9, baseline-corrected non-neutral expressiveness clustered around zero during the emotional demand scene, was reduced during the cognitive demand scene, and increased markedly during the physical and dual demand scenes, with the largest median change and greatest inter-individual variability observed in the dual demand condition. This suppression of expressive activity under cognitive load, contrasted with the heightened expressiveness under physically and doubly demanding scenarios, is consistent with expected patterns of facial stillness during focused concentration and greater expressive engagement when tasks are more embodied or multimodal. Overall, these patterns indicate that the CNN–TCN model captures meaningful, scene-dependent variations in facial expressiveness even in the absence of labeled data.

**Figure 10:**
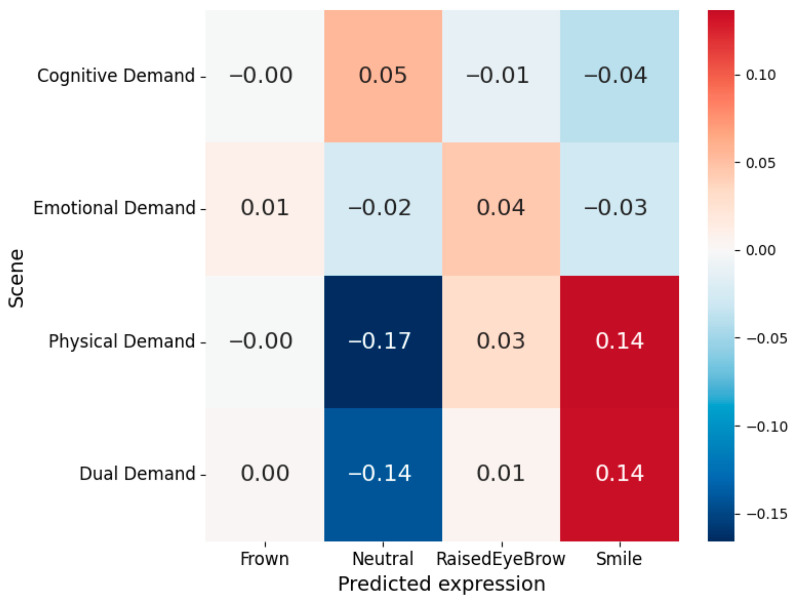
Mean change in predicted expression probability (Δ relative to baseline condition) for each VR scene. Values represent scene-level differences in average predicted expression proportions across participants. Positive values (red) indicate increased prevalence of the expression relative to baseline, whereas negative values (blue) indicate reduced prevalence.

To determine which expression categories drove these changes, for each trial and scene we computed the proportion of time classified as smile, frown, raised eyebrow, or neutral and subtracted that trial’s baseline proportions; the scene-averaged differences (Δ proportion vs. baseline) are shown in Figure 10. Relative to baseline, the physical demand scene exhibited the largest reduction in neutral predictions (−0.17) together with a pronounced increase in smile (+0.14) and a smaller increase in raised eyebrow (+0.03), indicating that enhanced expressiveness in this condition is primarily driven by positive-valence mouth and brow activity. The dual demand scene showed a similar pattern, with a marked decrease in neutral (−0.14) and a corresponding increase in smile (+0.14), while emotional demand produced only subtle shifts (slight decrease in neutral and small increase in raised eyebrow, all |Δ| ≤ 0.04). In contrast, the cognitive demand scene displayed a modest increase in neutral (Δ = +0.05) accompanied by small decreases in smile and raised eyebrow, reinforcing the interpretation that participants maintained a relatively neutral, still facial posture during cognitively demanding tasks. Across all scenes, frown probabilities remained close to zero, suggesting that negative-valence brow contractions were rare in this protocol.

**Figure 11:**
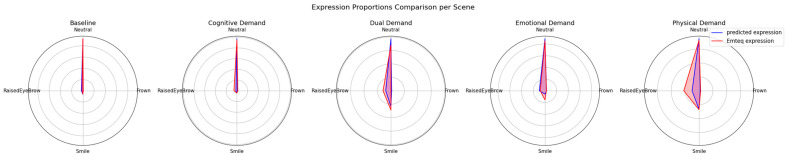
Mean expression proportions across VR scenes for the CNN–TCN model (blue) and Emteq-derived expression indices (red). Values represent average expression prevalence across participants within each scene. Radar axes correspond to neutral, frown, raised eyebrow, and smile categories. The figure illustrates relative differences in expression distributions between systems across experimental conditions.

To evaluate the physiological plausibility of the model’s predictions during the inference phase, the scene-level distribution of predicted expressions was directly compared against Emteq’s native expression indices. Figure 11 presents radar plots of expression proportions for each VR scene, showing side-by-side the CNN–TCN model outputs (blue) and the corresponding Emteq-derived expression estimates (red). Across all scenes, the model closely reproduced the dominant expressive pattern observed in the Emteq signal, namely, a strong predominance of neutral activity at baseline, modest increases in positive-valence expressions (smile and raised eyebrow) during the emotional and physical demand scenes, and minimal expressive output during the cognitive demand condition. The overlap of the blue and red indicates a high degree of correspondence, with the model tracking relative changes in scene-dependent expressiveness even though it was trained exclusively on calibration expressions and receives no ground-truth labels during the experimental session.

### 3.4. Scene-Dependent Associations Between Facial Expression Dynamics and Workload

To characterize the overall structure of the expression-derived feature space, we performed principal component analysis (PCA) on the 81 standardized expression features (see Table 3) and retained the first two components, which together explained 41.6% of the variance (PC1: 24.5%, PC2: 17.1%). Inspection of the PC1 loadings showed that this component was dominated by global expressiveness metrics, including entropy, switch rate, neutral and raised eyebrow burst counts, and transitions between neutral, smile, and raised eyebrow states (e.g., entropy, switch rate, neutral burst count, raised eyebrow burst count, neutral to smile). PC1 therefore captures a general “expression dynamics” axis, reflecting how frequently participants moved away from a stable neutral face into any non-neutral expression. PC2, in contrast, was defined primarily by frown-related features (e.g., count frown, frown burst mean/median/max, frown burst count, frown ratio) together with smaller contributions from surprised and eyebrow-burst variability, indicating that it indexes a tension/frustration dimension driven by corrugator-like activity rather than positive valence.

**Figure 12:**
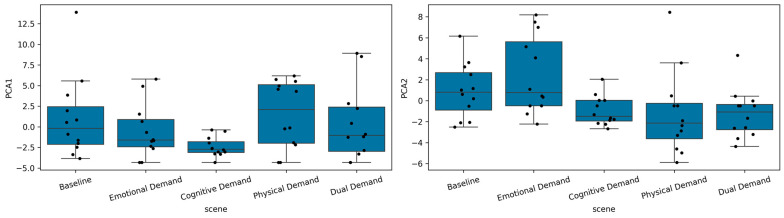
Distribution of participant-level principal component scores across VR scenes. PCA was performed on the full set of expression features, yielding two dominant components: PC1 (primarily reflecting dynamic expression transitions and variability) and PC2 (primarily reflecting frown-related tension features). Boxplots display the median and interquartile range of standardized PCA scores across participants within each scene; individual dots represent participant-level values. Black dots represent individual participant observations.

Scene-wise boxplots of PC1 and PC2 (Figure 12) and mixed-effects models with participant as a random intercept confirmed that these components varied systematically across conditions. For PC1, only the cognitive demand scene differed significantly from baseline (β = −3.93, *p* = 0.005), showing markedly reduced expression dynamics, whereas emotional, physical, and dual demand scenes did not significantly depart from baseline, consistent with the impression that cognitive load suppressed overall facial expressiveness. For PC2, emotional demand elicited significantly higher scores than baseline (β = 2.34, *p* = 0.031), whereas physical and dual demand scenes showed significantly lower scores (β = −2.23, *p* = 0.040; β = −2.31, *p* = 0.033), indicating that the emotionally evocative scene was associated with more frown-related tension, while the physically and dual-demand scenes were characterized by reduced frown activity. We performed this PCA step to reduce the high-dimensional feature set to a small number of interpretable latent dimensions that summarize global expressiveness (PC1) and frown-related tension (PC2). This allowed us to demonstrate that the model-derived expression features carry coherent, scene-dependent structure before moving to more detailed, feature-level and NASA-TLX-based mixed-effects analyses.

Building on the PCA structure, we next examined how individual expression features relate to subjective workload across scenes. To do so, we fitted a series of linear mixed-effects models in which each z-scored expression features predicted NASA-TLX ratings, with scene included as a fixed effect and an interaction term allowing feature–workload associations to vary by VR context. Participant-level random intercepts accounted for repeated measures and inter-individual differences in baseline workload. Models were estimated using restricted maximum likelihood with the Powell optimization algorithm, which yielded stable convergence across features. This modeling framework enabled us to quantify not only whether specific expressive behaviors were associated with subjective workload, but also whether the strength or direction of these associations was selectively modulated by task demands.

To facilitate interpretation, the extracted expression features were designed to capture both tonic and temporal characteristics of facial expression behavior within each scene. Specifically, the feature set includes summary descriptors of overall expression prevalence (e.g., ratios, counts, and burst-related measures) as well as sequence-level descriptors that reflect temporal organization (e.g., transition probabilities, latency indices, and entropy). All statistical inferences reported below are based on mixed-effects models with Benjamini–Hochberg FDR correction, and only associations surviving q < 0.05 are described (β coefficients and SE are reported for the fixed effects). Note that all reported relationships between facial expression dynamics and NASA-TLX dimensions are correlational in nature and should be interpreted as evidence of covariation and convergent validity rather than causal effects, due to the non-casual nature of regression analysis. Moreover, all expression predictors were z-scored prior to modeling; therefore, the reported β coefficients represent the change in NASA-TLX score (0–10 scale) per one standard deviation increase in the expression feature, enabling standardized comparison of effect sizes across features within each NASA-TLX dimension.

The CNN–TCN architecture served as the primary spatio-temporal modeling framework in this study, with subsequent benchmarking confirming its strong performance under LOPO cross-validation. The most consistent pattern—observed across both the CNN–TCN–derived features and Emteq-derived metrics—was a negative association between NASA-TLX physical demand and smile-related dynamics, indicating reduced smile persistence/expressivity under higher perceived physical workload. This convergent effect was observed across multiple dynamic and statistical smile-related features and across both modeling pipelines, suggesting a robust relationship between increased physical workload and reduced smile-related activity (broadly compatible with reduced FACS AU12-related engagement).

Additional FDR-surviving associations were observed between workload dimensions and eyebrow- and smile-related temporal dynamics in the emotional- and physical-demand scenes; however, these effects were less consistent across feature types and contexts. Overall, robust effects concentrated primarily on smile-related dynamics, with more selective modulation observed in eyebrow-related transitions. These effects may reflect modulation of expressive dynamics under affective challenge, though we interpret them cautiously given the exploratory, correlational nature of the analysis. Given the relatively small sample size and the number of tested associations across features and workload dimensions, all mixed-effects analyses reported here should be interpreted as exploratory and hypothesis-generating rather than confirmatory. Although FDR correction was applied to mitigate multiple-comparisons risk, these findings require replication in larger independent cohorts before firm conclusions can be drawn regarding the stability and generalizability of the observed relationships. Detailed feature-level statistics for all mixed-effects models are provided as (Appendix A.7).

### 3.5. Model Comparison

To contextualize the performance and interpretability of the proposed CNN-TCN framework, we conducted a complementary comparison. We benchmarked the CNN–TCN model against alternative ML architectures (CNN, TCN, LSTM, CNN-LSTM and GRU) which are commonly used for affective computing and physiological time-series analysis. While traditional fEMG studies frequently rely on handcrafted feature extraction followed by classical classifiers (e.g., SVM, LDA), the present framework operates directly on raw fEMG time-series and learns spatio-temporal representations end-to-end. Implementing a feature-based baseline would require constructing a separate feature-engineering pipeline distinct from the raw-input modeling strategy adopted here. Therefore, we evaluated representative spatial (CNN), temporal (TCN, LSTM, GRU), and hybrid (CNN–LSTM) architectures under identical LOPO conditions within a unified raw-input framework.

For this purpose, all neural architectures were evaluated exclusively under supervised conditions using participant-specific calibration data with ground-truth expression labels. As such, the deep learning comparison reflects relative model capacity under controlled supervision rather than end-to-end deployment performance. As summarized in Table 4, our proposed CNN–TCN achieves the strongest overall performance in terms of both Macro F1-score (0.882 ± 0.135) and Macro Recall (0.896 ± 0.107), indicating the most balanced sensitivity across expression classes under class imbalance. While the CNN baseline performs competitively, reflecting its effectiveness in capturing localized muscular activation patterns, its lower Macro F1 compared to CNN–TCN suggests limited access to longer-range temporal structure. In contrast, the TCN and recurrent models (LSTM, CNN–LSTM, GRU), which primarily emphasize temporal dependencies, exhibit reduced performance, likely due to weaker spatial feature extraction from fEMG signals. These results indicate that jointly modeling spatial muscle activations and long-range temporal dynamics, as realized by CNN–TCN, provides the most effective representation for calibrated facial expression learning in this dataset. Moreover, a closely related study using the same emteqPRO facial mask [64] reported an F1-Macro score of 0.86 for five posed expressions (smile, frown, raised eyebrow, squeezed eyes, neutral) using handcrafted feature extraction. In contrast, our proposed CNN–TCN model achieved a slightly higher Macro F1-score of 0.882 ± 0.135 under LOPO cross-validation, while operating directly on raw fEMG time-series without manual feature engineering. Although the prior study included an additional “squeezed eyes” class, which increases classification complexity due to muscle overlap with frown, the comparable performance achieved by the end-to-end CNN–TCN model suggests that learned spatio-temporal representations can match or exceed feature-based pipelines on the same sensing hardware.

## 4. Discussion

The results confirm that the experimental protocol successfully elicited distinct emotional and workload states across VR scenes, as supported by both self-reports and continuous facial expression data. Emoji-based mood ratings and NASA–TLX scores showed significant scene-dependent variation, indicating effective manipulation of emotional and cognitive demands. In parallel, spontaneous fEMG activity exhibited physiologically coherent, muscle-specific modulation across scenes, supporting the validity of linking facial muscle dynamics with task context. Together, these findings demonstrate the feasibility of combining immersive VR with wearable fEMG to investigate cognitive–emotional dynamics in ecologically valid conditions.

Although self-report measures captured broad differences between scenes, they provided limited resolution for complex affective states influenced by stress, effort, and engagement. The convergence between workload ratings and fEMG-derived expression dynamics highlights the value of multimodal approaches for capturing subtle cognitive–emotional variations. Incorporating more granular affective scales and additional physiological modalities in future work may further improve sensitivity to nuanced emotional states.

These findings build on prior work demonstrating the feasibility of integrating facial EMG into immersive VR systems and using it for affect and expression monitoring [52,53,55,65,66,67]. While most previous fEMG–VR research has focused on discrete emotions or short events, relatively few studies have examined continuous expressive dynamics during cognitively demanding VR scenarios. The present work addresses this gap by characterizing temporally evolving expression patterns associated with workload and emotional context in immersive environments.

The proposed CNN–TCN framework achieved robust expression classification and generalized across participants and VR scenes, supporting the value of spatio-temporal deep learning for modeling coordinated facial muscle activity. This approach, through a calibration session where participants were asked to make intentional facial expressions, proved very promising despite being trained on only 12 participants. Our CNN-TCN model exhibited strong performance in detecting facial expressions, with high classification reliability. This finding is particularly noteworthy when comparing our results with Emteq’s native expression indices. Compared with traditional handcrafted feature pipelines and classical classifiers [65,68,69], deep-learning approaches better capture complex spatial and temporal dependencies in multi-channel fEMG [36,66,70,71,72]. We further evaluated transfer by applying the trained model to experimental fEMG recordings containing spontaneous, VR task-elicited expressions, and validated the dynamics through physiological plausibility and convergent associations with NASA-TLX workload measures. Scene-independent inference revealed physiologically meaningful variations in expression dynamics, and mixed-effects analyses identified statistically robust associations between expression-derived features and subjective workload, with FDR-surviving effects primarily related to perceived physical demand. These findings support the potential of deep-learning–based fEMG analysis as a continuous and objective complement to intermittent self-report measures in immersive VR.

While the results are promising, several limitations should be acknowledged. This initial pilot study is limited by its small sample size and the specificity of the VR tasks, which constrain the extent to which findings can be generalized beyond the current cohort. Although LOPO cross-validation, high-resolution windowed fEMG data, and regularized spatio-temporal modeling were employed to mitigate overfitting, the results should be interpreted as cross-validated within this sample rather than evidence of broad population-level generalization. External validation on larger and more diverse cohorts, including variation in age, ethnicity, and facial morphology, will be necessary before supporting wider deployment.

The facial expressions used in this study were limited to four expressions (smile, frown, raised eyebrow, and neutral), dictated by the standardized Emteq calibration protocol [52], and employed as expression-level markers rather than direct representations of complex emotional states. Accordingly, the present framework should be interpreted as a proof-of-concept for mapping wearable fEMG-derived expression dynamics to cognitive workload, rather than as a comprehensive emotion recognition system. While we have implemented controlled conditions and advanced signal processing techniques to mitigate non-emotional influences, we acknowledge that factors such as physical exertion, breathing patterns, and mechanical artifacts due to movement within the immersive environment may still affect fEMG signals, particularly during physical and dual-task VR scenes. Future extensions incorporating richer expression vocabularies and multimodal signals may enable more direct modeling of higher-level affective constructs. Such extensions could include FACS-informed labeling of additional facial action units, expanded calibration tasks designed to elicit a broader range of expressions, and transferring learning approaches leveraging larger facial expression datasets to improve robustness and generalizability across users and contexts. In addition, incorporating more granular emotion scales, such as the Self-Assessment Manikin (SAM) [73] or the Positive and Negative Affect Schedule (PANAS) [74], could enhance our ability to capture the subtle complexities of emotional states, providing a more comprehensive understanding of the interplay between emotional experiences and experimental conditions. Furthermore, a formal a priori power analysis was not conducted, given the exploratory nature of the study and the lack of established effect size estimates for CNN–TCN–based fEMG modeling in immersive VR.

Future research will focus on expanding participant diversity, evaluating generalization across broader VR contexts, and transitioning toward real-time deployment. Integrating additional physiological signals and optimizing streaming inference will be essential for scalable implementation. Potential applications include adaptive training environments, therapeutic VR, and user-experience assessment, provided that appropriate safeguards for biometric data privacy and ethical use are maintained.

## 5. Conclusions

This study investigated whether multi-channel fEMG, combined with spatio-temporal deep learning, can support participant-shared expression modeling and its transfer to spontaneous behavior in immersive VR. Using participant-level physiological normalization and a single shared CNN–TCN architecture, we achieved strong leave-one-participant-out classification performance for four calibrated facial expressions. When applied to unlabeled VR task-elicited fEMG recordings, the trained model generated continuous expression dynamics that varied systematically across scenes. Derived static and temporal features showed FDR-surviving associations primarily with perceived physical demand (NASA-TLX), and the observed activation patterns were physiologically plausible and consistent with FACS-based interpretations. Together, these findings demonstrate that end-to-end modeling of raw fEMG signals can bridge brief calibration-based learning and spontaneous task-elicited facial behavior in immersive environments. By avoiding handcrafted feature engineering and relying on a shared model trained across participants, the framework supports privacy-preserving, physiologically grounded expression sensing in VR. Importantly, the results should be interpreted as pilot-level evidence, given the limited sample size. Several avenues for future work emerge from this study. Expanding to larger and more diverse cohorts will strengthen generalizability and statistical power. Incorporating additional expression categories and multimodal physiological signals may enable richer modeling of affective and workload-related constructs. Investigating few-shot adaptation or alternative normalization strategies could reduce reliance on participant-level calibration. Finally, addressing real-time deployment challenges, such as latency optimization, buffering strategies, and adaptive model updating, will be essential for translating this framework into practical applications in training, therapy, and human performance monitoring.

## Figures and Tables

**Table 1:** Primary facial muscles involved for a specific facial expression according to the FACS.

Emotion (FACS)	Expression (Emteq)	Frontalis	Corrugator	Orbicularis	Zygomaticus
Happiness	Smile			×	×
Sadness	Frown	×	×		
Surprise	Eyebrow Raising	×			
Fear		×	×	×	
Anger			×	×	
Disgust				×	
Contempt					×

Note. FACS = Facial Action Coding System. Emteq refers to the facial EMG–based expression categories provided by the Emteq system. Crosses (×) indicate primary involvement of the corresponding muscle in the expression.

**Table 2:** Average classification performance for facial expression of the CNN–TCN model across all LOPO cross-validation folds.

Metric	Train	Validation	Test
Accuracy	0.9616 ± 0.0149	0.9352 ± 0.0704	0.9306 ± 0.0716
Macro F1-score	0.9554 ± 0.0216	0.8873 ± 0.1355	0.8822 ± 0.1346
Weighted F1 -score	0.9608 ± 0.0154	0.918 ± 0.0957	0.9133 ± 0.0959
Macro Precision	0.9725 ± 0.0202	0.8938 ± 0.1533	0.8920 ± 0.1525
Weighted Precision	0.9633 ± 0.015	0.9167 ± 0.112	0.9133 ± 0.1115
Macro Recall	0.9424 ± 0.0223	0.9028 ± 0.1056	0.8958 ± 0.1073
Weighted Recall	0.9616 ± 0.0149	0.9352 ± 0.0704	0.9306 ± 0.0716
ROC AUC	0.9881 ± 0.0058	0.9795 ± 0.0468	0.9564 ± 0.0646

Note. Values are reported as mean ± standard deviation across LOPO cross-validation folds. Macro and weighted metrics are computed by averaging class-wise and class-frequency–weighted scores, respectively. ROC AUC = Receiver Operating Characteristic Area Under the Curve; F1 = F1-score.

**Table 3:** Overview of facial expression–derived temporal and statistical features (# denotes the number of features.).

Category	Feature Type	# Features	Coverage	Description
Expression Ratios	Ratio (0–1)	4	Neutral, Frown, Smile, Raised Eyebrow	Proportion of time spent in each expression state.
Expression Counts	Count	5	Same 4 states + total frames	Raw counts of frames labeled with each expression and total count.
Neutral → Expression Transitions	Count	3	Neutral → (Frown, Smile, Raised Eyebrow)	How often does neutral transition into each non-neutral expression.
Average Burst Lengths	Mean	3	Frown, Smile, Raised Eyebrow	The mean duration of continuous expression bursts before switching.
Entropy Metrics	Score	2	Full sequence	Shannon entropy and normalized entropy capturing variability.
Sequence Dynamics	Count/Rate/Ratio	3	Full sequence	Global temporal dynamics: switches, switching rate, and persistence.
Dominance and Balance	Ratio/Inequality Score	2	Non-neutral expressions	Distribution of balance of non-neutral expressions (dominance and Gini-like inequality).
Latency After Neutral	Index	3	Frown, Smile, Raised Eyebrow	Latency until the first occurrence of each expression after neutral.
Transition Counts (A → B)	Count	16	All 4 × 4 transitions between states	Count of transitions between every pair of expressions (16 total).
Transition Probabilities (A → B)	Probability	16	All 4 × 4 transitions between states	Normalized probability of transitioning from each expression to the next.
Burst Statistics	Count	4	Neutral, Frown, Smile, Raised Eyebrow	Distributional descriptors of burst durations for each facial expression.

**Table 4:** LOPO performance comparison of DL models on calibrated expression labels.

Metrics	CNN	TCN	LSTM	CNN-LSTM	GRU	CNN-TCN
Macro F1-score	0.8326 ± 0.1307	0.8008 ± 0.2136	0.8102 ± 0.1307	0.7607 ± 0.1626	0.7438 ± 0.1679	**0.8822 ± 0.1346**
Macro Recall	0.8588 ± 0.0994	0.8449 ± 0.1600	0.8495 ± 0.1082	0.7894 ± 0.1453	0.8032 ± 0.1332	**0.8958 ± 0.1073**

Note. Values are reported as mean ± standard deviation across Leave-One-Participant-Out (LOPO) cross-validation folds. CNN = Convolutional Neural Network; TCN = Temporal Convolutional Network; LSTM = Long Short-Term Memory; GRU = Gated Recurrent Unit. Bold values indicate the best performance among the compared models for each metric.

**Figure A1:**
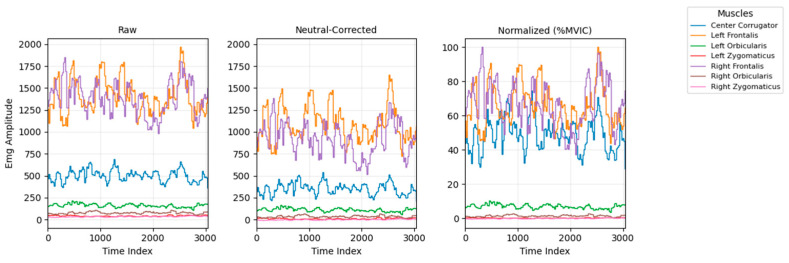
Preprocessing transformation of fEMG signals from example participant illustrated on raised eyebrow calibration data: raw, neutral-corrected, and %MVIC-normalized activity.

**Figure A2:**
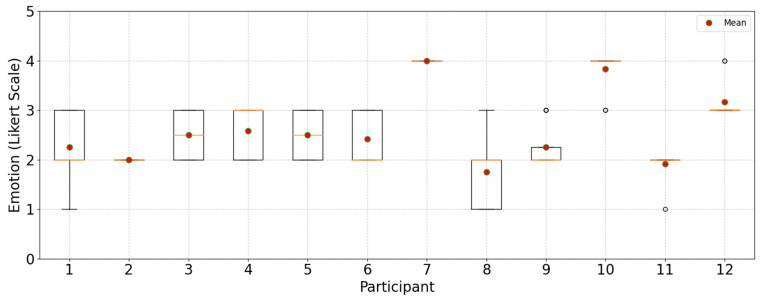
Average emotion ratings over all scenes by participants. In cases where the box collapses into a single line, this indicates no observed variability in the participant’s ratings across scenes.

**Figure A3:**
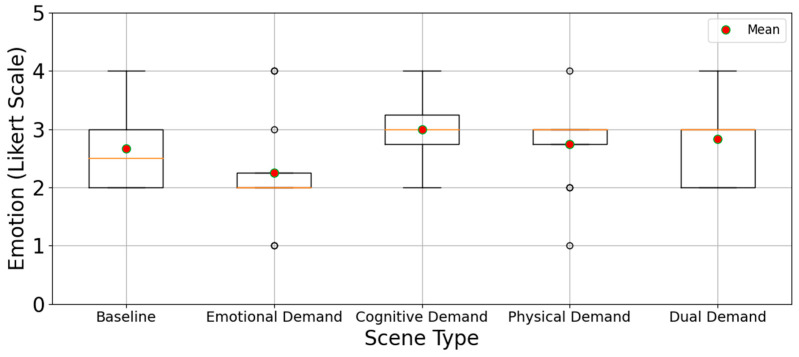
Average emotion ratings by scene.

**Figure A4:**
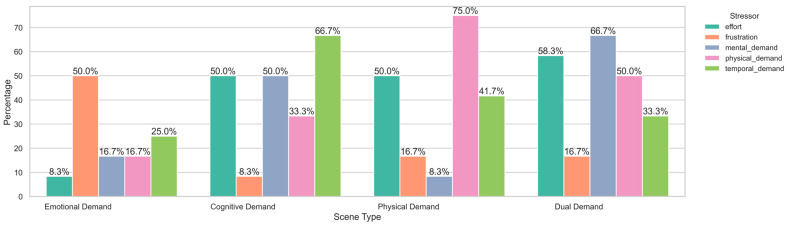
Dominant stressors identified by NASA-TLX ratings. VR exercises targeted cognitive, emotional, physical, and dual demands, measured across six dimensions in legend: effort, frustration, mental, physical, and temporal demand. A threshold of ≥5 identified dominant stressors per scene. Performance ratings were verified but excluded from analysis. The figure shows sub-items exceeding this threshold for each scene.

**Figure A5:**
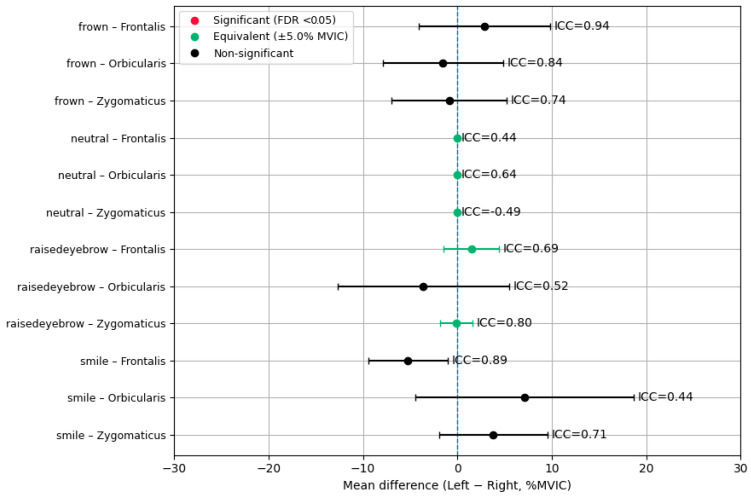
Left–right symmetry of normalized fEMG activity across facial expressions and muscles. Points represent the mean difference in activation between the left and right sides (%MVIC), with error bars indicating variability across participants. Colors denote statistical interpretation: red = significant difference (FDR < 0.05), green = equivalence within ±5.0% MVIC, and black = non-significant difference. The absence of significant differences (red) suggest that there is bilateral symmetry in the examined muscles.

**Figure A6:**
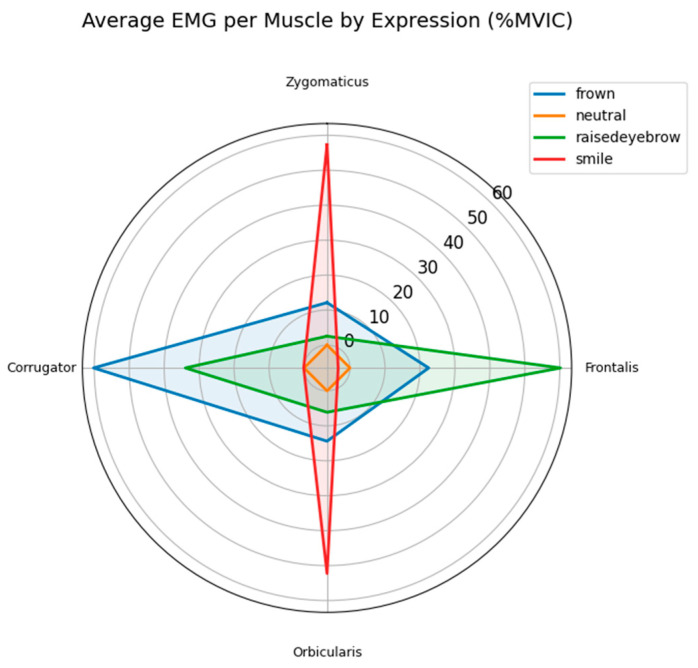
Radar plot illustrating the %MVIC across the four facial muscles, highlighting distinct and physiologically coherent activation patterns for each expression. MVIC = Maximum Voluntary Isometric Contraction.

**Figure A7:**
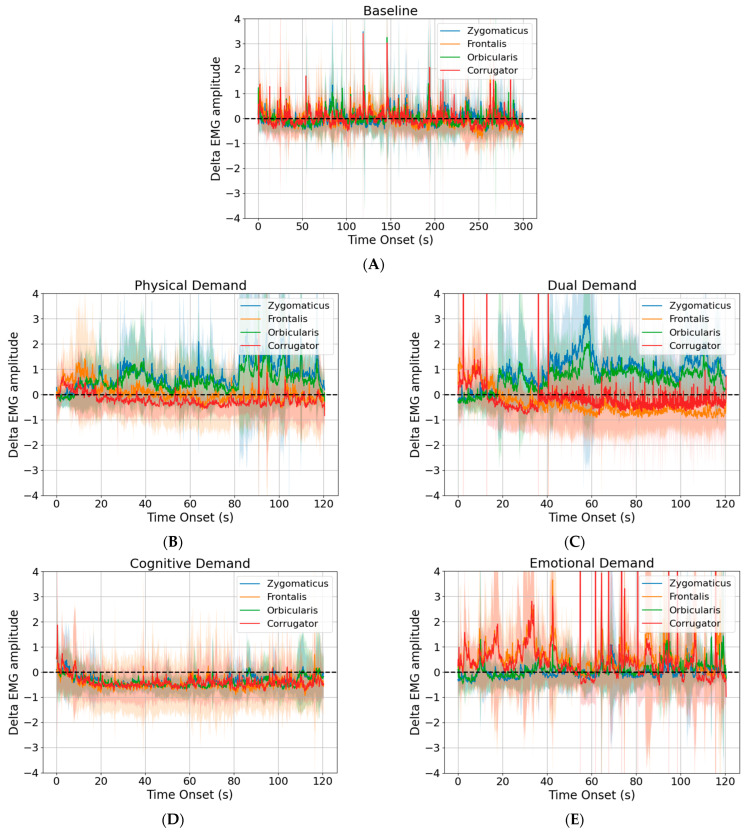
Mean normalized fEMG amplitude (Δ relative to baseline, MVIC-scaled) across participants for each VR scene. Signals are aligned to scene onset (time = 0 s) and averaged across participants. Solid lines represent the group mean; shaded regions indicate ±1 standard deviation. Channels correspond to Zygomaticus, Frontalis, Orbicularis, and Corrugator. The dashed horizontal line denotes zero-centered baseline activity. Signals collected from (**A**) baseline, and scenes with task demands, (**B**) physical, (**C**) dual, (**D**) cognitive and (**E**) emotional.

## Data Availability

The data presented in this study are not readily available due to ethical restrictions preventing sharing with investigators outside this study. Access to the dataset may be made available upon request by contacting the corresponding author.

## Abbreviations

The following abbreviations are used in this manuscript:

ANOVA	Analysis of Variance
AR	Autoregressive
AU	Action Unit
BN	Batch Normalization
CI	Confidence Intervals
CNN-TCN	Convolutional Neural Networks and Temporal Convolutional Networks
DL	Deep Learning
DRDC	Defence Research and Development Canada
EEG	Electroencephalography
FACS	Facial Action Coding System
FC	Fully Connected
FDR	False Discovery Rate
fEMG	Facial Electromyography
GRU	Gated Recurrent Unit
HMM	Hidden Markov Model
HR	Heart Rate
HREC	Human Research Ethics Committee
HRV	Heart Rate Variability
ICC	Intraclass Correlation Coefficient
KNN	k-Nearest Neighbors
LDA	Linear Discriminant Analysis
LOPO	Leave-One-Participant-Out
MAV	Mean Absolute Value
ML	Machine Learning
MVIC	Maximum Voluntary Isometric Contraction
NASA-TLX	NASA Task Load Index
NRC	National Research Council Canada
PCA	Principle Component Analysis
PC	Principle Component
PSD	Power Spectral Density
REB	Research Ethics Board
RMS	Root Mean Square
ROC-AUC	Receiver Operating Characteristic Area-Under-the-Curve
STDF	Spatio-Temporal Deep Forest
SVM	Support Vector Machines
TCN	Temporal Convolutional Network
TOST	Two One-Sided Test
VR	Virtual Reality

## Appendix A

### Appendix A.1. Signal Normalization Procedure

This appendix describes the two-stage normalization pipeline applied to the fEMG signals and illustrates their effects using representative calibration data. Figure A1 shows the impact of each normalization step on raised eyebrow calibration recordings from a representative participant across all facial muscle channels.

Neutral Correction: For each participant and each muscle channel, the mean fEMG amplitude was computed from that participant’s neutral calibration segments. These participant-specific neutral values were then subtracted from all calibration and experimental fEMG recordings to perform neutral correction. As shown in the middle panel of Figure A1, this procedure removes participant-specific resting-tone differences and low-frequency baseline offsets: the raw signals (left panel) exhibit large variations in baseline amplitude across channels, whereas the neutral-corrected signals oscillate around zero.

MVIC Normalization: This scales the muscle activation relative to each individual’s maximum capacity, ensuring that amplitude differences are minimized across participants. This step is vital for ensuring that the fEMG amplitudes are comparable across individuals, as it accounts for physiological differences in muscle activation capabilities. MVIC represents the maximum muscle activation a participant can voluntarily generate under standardized conditions and is commonly used to normalize fEMG amplitudes across individuals [12]. For each muscle channel, the MVIC value was derived from the calibration expression that maximally activates that muscle: zygomaticus (smile), corrugator (frown), and frontalis (eyebrow-raise). Because we did not include a dedicated calibration task to maximally activate the orbicularis muscle, the MVIC for this channel was defined as the maximum observed activation across all calibration expressions. The peak activation obtained from these calibration trials was taken as the MVIC for that muscle. All calibration and experimental signals were then normalized by dividing by the MVIC and multiplying by 100, yielding fEMG amplitudes expressed as a percentage of each participant’s maximum voluntary activation [12]. As illustrated in the right panel of Figure A1, MVIC normalization maps all channels onto a consistent physiological scale, reducing amplitude disparities between muscles and across participants.

### Appendix A.2. Model Training Details

The CNN–TCN expression classifier was trained using a multiclass cross-entropy loss function optimized with the Adam optimizer (learning rate = 5 × 10^−4^) and a batch size of 128. Early stopping with a patience of three epochs was applied to ensure stable convergence and mitigate overfitting. All experiments were conducted using a fixed random seed (42) to support reproducibility. Calibration signals were segmented into non-overlapping 1 s windows to minimize temporal redundancy and reduce overfitting caused by highly correlated overlapping samples. The same windowing strategy was applied during inference to ensure consistency between training and application. Because the calibration dataset was imbalanced, particularly due to a large proportion of neutral expression segments, model performance was quantified using both weighted and macro-averaged evaluation metrics to ensure that minority expression classes contributed proportionally to the overall assessment. The components of the CNN–TCN model are described below.

**CNN Block:** The CNN block consists of two consecutive 1D convolutional layers with 32 filters each (kernel size = 5, stride = 1, padding = 2) followed by Batch Normalization (BN) and Rectified Linear Unit (ReLU) activation. This configuration was selected empirically through experimental evaluation of different model structures and hyperparameter settings. CNNs are effective in identifying short-term spatio-temporal features (such as bursts or peaks) and provide translation invariance across time. The first layer extracts localized temporal features across channels (e.g., muscle activation bursts). The second layer refines these patterns by combining nearby temporal dependencies and enhancing robustness to noise.

**TCN Block:** TCNs are well-suited for sequential physiological data, offering parallel computation, stable gradients, and flexible receptive fields compared to recurrent architectures such as long short-term memory (LSTMs) [75]. The extracted feature maps are then passed through the TCN network. The TCN block used a kernel size of 3 and comprised three residual blocks with channel sizes of 32, 32, and 64, and dilation factors of 1, 2, and 4, respectively. Each residual block contains two dilated convolutions with dropout = 0.2 and skips connections, allowing the model to capture long-range temporal dependencies without losing fine temporal resolution. All convolutional layers used stride = 1 and zero padding to preserve temporal resolution.

**FC Block:** Following temporal feature extraction, an adaptive average pooling layer reduced the temporal dimension before a fully connected layer of 128 units. A ReLU activation and dropout (*p* = 0.2) were applied prior to the final linear classification layer producing logits for the four expression classes. Then a softmax activation is used at the last layer of the FC block to label the fEMG signals.

The model was trained using the Adam optimizer with a learning rate of 5 × 10^−4^ and a batch size of 128, minimizing the multiclass cross-entropy loss function. Early stopping was implemented based on validation loss with a patience of three epochs to prevent overfitting and ensure stable convergence. For data preprocessing, calibration signals were segmented into non-overlapping 1 s windows, and the same windowing strategy was applied during inference to maintain methodological consistency between training and deployment phases.

### Appendix A.3. Features Description

After generating continuous time-series of predicted expressions in the inference phase, we aim to go beyond expression classification and investigate whether facial expression dynamics could reveal more nuanced patterns related to cognitive, emotional, physical, and mental workload. To do so, a comprehensive set of expression-based features is computed from the predicted facial expression time-series.

Comprehensive list of features and their categories:
Composition: Ratios and counts of frown, smile, raised eyebrow, and neutral expressions (e.g., smile ratio, frown ratio, count smile);
Dynamics: Transition counts and probabilities between expression states, including both neutral-to-expression transitions and full 4 × 4 transition matrices (e.g., neutral to frown, transition smile to frown probability);
Persistence: Burst-based metrics capturing how long each expression is sustained, including mean, median, maximum duration, standard deviation, and coefficient of variation (e.g., smile burst mean, frown burst max);
Variability: Measures of sequence complexity such as entropy and normalized entropy (e.g., entropy, normalized entropy), as well as switching rates (switch rate, persistence rate).

Across these categories, the full feature set included ratios, counts, burst statistics, full transition matrices, latency indices, dominance measures, and entropy-based metrics.

### Appendix A.4. Verification of Emotion Elicitation

The purpose of this study is to establish the relationship between variations in psychophysiological signals and the corresponding emotional state triggering these changes. To achieve this, the experimental procedure was meticulously designed to evoke targeted emotional responses. As a first step, it was verified that the intended emotions were indeed elicited. Participants were asked to assess their emotional states, both during the baseline and during the task-based scenes, as a general reflection on their perception of the stimuli. To capture both basic and more nuanced emotions, two types of self-report measures were used. Additionally, facial expression data were captured continuously during both the calibration and experiment testing phases and subsequently evaluated. Taken together, it appears that the experimental procedure was adequate, since all the intended responses appear to have been triggered during the experiment.

#### Appendix A.4.1. Basic Emotional Response—Self-Reported via Likert Scale

The self-reported mood ratings collected via the emoji-based Likert scale (0–5) showed that most participants exhibited discernible variability in emotional ratings across the experimental scenes, while only two participants (#2 and #7) reported the same rating across all scenes, indicating limited response bias and successful differentiation in emotional experience across scenes. In the aggregated emotion ratings across the five scenes, as anticipated, participants’ mood ratings were generally lower relative to baseline in the emotional scene, designed to induce frustration. In contrast, scenes incorporating task stressors (e.g., cognitively demanding and dual) prompted higher self-reported emotion scores, pointing to stronger engagement or arousal under cognitive load. These nuanced emotions are influenced by factors like mental effort and stress, which may not be fully captured by a simple mood rating scale. This suggests that while basic emotions can be adequately captured using a mood rating, more nuanced emotions, such as stress from task load, may require alternate tools for comprehensive understanding. The self-reported mood ratings collected via the emoji-based Likert scale (0–5) are presented in Figure A2. Figure A3 shows the aggregated emotion ratings across the five scenes, illustrating both the mean (red) and median (blue) values with interquartile ranges.

A Friedman test for repeated measures was conducted to evaluate whether the median emotion ratings differed across scenes. The analysis revealed a significant main effect of scene, χ^2^(4) = 10.93, *p* = 0.027. The corresponding Kendall’s W = 0.23 indicated a small-to-moderate effect size, suggesting that approximately 23% of the variance in ranked emotion scores was attributable to differences in scene context. Post hoc Conover pairwise comparisons with Holm correction confirmed that only the emotional vs. cognitive demanding contrast reached significance ($p_{adj}$ = 0.011), whereas all other pairwise differences were non-significant ($p_{adj}$ ≥ 0.146). Inspection of medians showed that emotion ratings were lowest in the emotional scene and highest in the cognitive demanding scene, consistent with the intended experimental inductions of frustration and engagement, respectively. These findings indicate that participants’ self-reported emotional states varied systematically across scenes, validating the scene-induced variation in mood and demonstrating that the emoji-based Likert measure was sensitive to changes in affective state across different experimental conditions.

#### Appendix A.4.2. Nuanced Emotions: Self-Reported via the NASA-TLX

We used NASA-TLX as a self-reported measure to ensure that the designed VR exercises targeted the intended type of demand leading to more nuanced emotions, that is, cognitive, emotional, physical, and dual. The NASA-TLX measures perceived workload across six dimensions: mental, physical, and temporal demands of a task, as well as perceived performance, effort, and frustration levels. A threshold was used, for each participant and scene; dimensions with the highest NASA-TLX rating or with a score ≥ 5 (on a 0–10 scale) were identified as the dominant stressors, indicating that the corresponding type of workload demand was being elicited. Since the study was designed to not elicit demand related to poor performance (a potential confound), for the performance dimension we simply verified that participants indeed perceived their performance as adequate across tasks but not intended for inclusion in the analyses. Finally, since all exercises invoked a type of demand except for the one involving emotional demand, they were expected to involve an element of effort.

All reports of a NASA-TLX sub-item exceeding the threshold were identified and plotted for each of the scenes, Figure A4. It was seen that each of the scenes targeted the intended and specific task demands, specifically: emotional demand scene (frustration), cognitive demand scene (temporal/cognitive/effort), physical demand scene (physical/effort) and the dual scene (mental/effort/physical/temporal). To examine whether the four VR scenes elicited distinct workload profiles, NASA–TLX subscale scores were analyzed using Friedman tests followed by Wilcoxon signed-rank tests with Holm correction for multiple comparisons. This non-parametric approach was selected because the ratings are ordinal, the design was fully within-subjects, and the small sample size and deviations from normality made parametric repeated-measures Analysis of Variance (ANOVA) assumptions difficult to justify.

Friedman tests indicated significant scene effects for mental, physical, temporal demand, performance, and effort, but not frustration (see Table A1). Post hoc Wilcoxon tests showed that mental demand was higher in the dual-task condition than in the purely physical condition, and that emotional demand scene was rated as less physically demanding than both cognitive and dual tasks, aligning with the intended task manipulations. Performance ratings for the emotional demand scene differed from other conditions, although participants generally reported adequate performance across all tasks. For temporal demand and effort, only the omnibus tests were significant, with no pairwise comparison surviving Holm correction, suggesting modest differences between scenes. Overall, the findings support the intended differentiation of workload types across scenes, while performance was primarily used as a manipulation check rather than a primary outcome in subsequent analyses.

**Table A1:** Friedman tests were used to compare NASA–TLX subscale ratings across four VR scenes. Post hoc Wilcoxon signed-rank tests were conducted only when the omnibus test was significant, and *p* values were adjusted using the Holm method.

NASA-TLX Subscale	Friedman χ^2^(3)	*p*	Significant Post Hoc Wilcoxon Comparisons (Holm-Adjusted *p*)
Mental demand	12.514	0.0058	physical vs. dual demand scenes ($p_{adj}$ = 0.0205)
Physical demand	15.971	0.0011	emotional vs. cognitive demand scenes ($p_{adj}$ = 0.0207); emotional vs. dual demand scenes ($p_{adj}$ = 0.0088)
Temporal demand	9.196	0.0268	— (No pairwise comparison survived Holm correction)
Performance	23.694	<0.0001	emotional vs. cognitive demand scenes ($p_{adj}$ = 0.0119); emotional vs. physical demand scenes ($p_{adj}$ = 0.0121); emotional vs. dual demand scenes ($p_{adj}$ = 0.0029)
Effort	16.676	0.0008	— (No pairwise comparison survived Holm correction)
Frustration	5.011	0.1710	— (omnibus test not significant)

Note. NASA-TLX = NASA Task Load Index. Friedman χ^2^(3) indicates the Friedman test statistic with three degrees of freedom. *p* = *p*-value of the omnibus Friedman test. Post hoc comparisons were conducted using pairwise Wilcoxon signed-rank tests with Holm correction for multiple comparisons. $p_{adj}$ = Holm-adjusted *p*-value. Dashes (—) indicate that no pairwise comparison remained significant after correction or that the omnibus test was not significant.

### Appendix A.5. Verification of fEMG Signals Signatures—Validation in Calibration Phase

For the fEMG signals in the calibration phase, we first established bilateral reliability. Since facial muscles are symmetrically organized and the subsequent statistical analyses rely on averaged left–right activity, it was essential to confirm that both sides produced comparable responses. We verified that the fEMG recordings were bilaterally consistent. Because facial muscles are symmetrically organized and the subsequent statistical analyses rely on averaged left–right activity, it was essential to confirm that both sides produced comparable responses. To this end, left–right fEMG symmetry was assessed for each expression and muscle using equivalence testing within a ±5%MVIC tolerance and intraclass correlation coefficients (ICCs) as reliability indices. As shown in Figure A5, the mean left–right differences were centered near zero across all expressions, with most pairs falling inside the predefined equivalence bounds. ICC values ranged from 0.44 to 0.94, reflecting moderate-to-high agreement between sides. Only a few muscle–expression pairs showed small, non-significant deviations, and no consistent lateral bias was observed. These findings indicate that the bilateral electrodes captured comparable signals on both sides of the face, allowing subsequent analyses to average left and right channels to obtain a single, stable estimate of each muscle’s activation.

Having established bilateral reliability, we proceeded to investigate the reliability of fEMG signals in capturing activation signatures when participants are instructed to perform specific facial expressions. We examined the overall activation profiles for each instructed expression. The radar plot in Figure A6 illustrates the %MVIC across the three bilateral muscles (Frontalis, Orbicularis, and Zygomaticus) and the Corrugator. Each expression produced a distinct and physiologically coherent activation pattern:

Smile elicited the strongest activity in the Zygomaticus and Orbicularis muscles, reflecting cheek elevation and peri-oral tension.

Frown was characterized by dominant Corrugator activation associated with brow contraction.

Raised-eyebrow expressions primarily engaged the Frontalis, consistent with forehead elevation.

Neutral trials showed minimal activation across all muscles, confirming an effective baseline.

These topographies confirm that the calibration tasks elicited the intended muscle groups and that the fEMG signals captured the canonical physiological signatures of each expression.

To quantify these patterns statistically, we compared each expression’s activation to its neutral using paired *t*-tests with false-discovery-rate correction. The results are summarized below (mean ± 95% CI %MVIC difference, $p_{FDR}$ < 0.05):

**Frown:** Corrugator +60.2% MVIC [47.1–73.2], $p_{FDR}=8\times 10^{-6}$, Cohen’s d = 2.94; Frontalis +22.5% MVIC [3.2–41.8], $p_{FDR}=1.3\times 10^{-2}$, Cohen’s d = 0.74; smaller increases were also observed in Orbicularis (+14.4%) and Zygomaticus (+12.1%). This pattern confirms strong, targeted activation of the Corrugator with moderate co-activation of adjacent regions.

**Raised-eyebrow:** Frontalis +60.2% MVIC [56.8–63.6], $p_{FDR}<10^{-12}$, Cohen’s d = 11.19; Corrugator +33.9% MVIC [18.6–49.3], $p_{FDR}=5\times 10^{-4}$, Cohen’s d = 1.40; smaller increases in Zygomaticus (+2.5%) and Orbicularis (+6.1%). This pattern demonstrates a clear isolation of the Frontalis as the primary muscle involved in brow elevation.

**Smile:** Zygomaticus +57.3% MVIC [50.3–64.3], $p_{FDR}=3.4\times 10^{-9}$, Cohen’s d = 5.17; and Orbicularis + 52.2% MVIC [42.9–61.6], $p_{FDR}=8.7\times 10^{-8}$, Cohen’s d = 3.56. Corrugator and Frontalis remained close to neutral levels. This result defines the characteristic zygomatic–orbicularis synergy underlying the smiling expression.

Overall, the results provide strong evidence that the calibration protocol and fEMG acquisition pipeline effectively isolate the characteristic muscle activations of each core facial expression, yielding distinct, reproducible, and physiologically meaningful fEMG signatures that form a reliable foundation for further behavioral and computational analyses.

### Appendix A.6. Verification of fEMG Signals Signatures in VR Stressor Scenes

To validate the use of CNN–TCN model in the experimental setting, we examined the fEMG signals recorded during the VR stressor sessions. The goal of this analysis was to determine whether spontaneous facial expressions exhibited distinct spatial and temporal activation patterns across scenes, thereby supporting the use of a model designed to capture both dimensions. As shown in Figure A7, the muscle activations during the experimental scenes demonstrated clear, scene-specific modulation patterns similar to those observed in the intentional calibration expressions.

Each muscle’s activity was normalized using participant-specific baseline statistics (mean and standard deviation), resulting in the baseline scene being centered around zero and indicating no net deviation in facial activation. Experimental scenes, by contrast, showed distinct muscle-specific deviations. For visualization of temporal fEMG dynamics (Figure A7), the y-axis represents the aggregated delta fEMG amplitude over all participants, computed as each muscle’s activation relative to its baseline (scene—baseline, in z-units). To emphasize meaningful physiological trends while minimizing the visual influence of rare motion-related spikes, the y-axis was clipped to ±4 z.

Overall, muscle activations were not continuously sustained but instead appeared as transient peaks that rose and returned to baseline over time. This behavior highlights the inherently dynamic nature of facial muscle responses and underscores the need for temporal modeling approaches such as CNN–TCN. Scene-specific patterns were consistent with expected emotional and cognitive demands. During the cognitive demand scene, all facial muscles exhibited slightly negative delta fEMG values, indicating reduced activation consistent with increased cognitive load and sustained focus. In the emotionally demanding scene, simultaneous elevations in corrugator and frontalis activity suggested combined frowning and eyebrow-raising responses associated with alertness, surprise, or mild frustration. The physical and dual scenes exhibited pronounced increases in zygomaticus and orbicularis activity, reflecting smile-related activation commonly associated with positive engagement, relief or strenuous effort. These temporal fEMG signatures demonstrate that the system reliably captured meaningful physiological responses aligned with the intended demands of each VR scene. To statistically evaluate spontaneous facial responses during the experimental session, bilateral fEMG activity was compared across scenes relative to each participant’s baseline. For each muscle, the left and right signals were averaged, and mean activation (%MVIC) was analyzed using paired-sample t-tests (scene > baseline). The resulting statistics, summarized in Table A2, include the mean activation difference (scene − baseline, %MVIC), 95% confidence intervals, t-values, Benjamini–Hochberg FDR-corrected *p*-values, and standardized effect sizes (dz, Cohen’s d for paired designs).

**Table A2:** Statistical evaluation of higher bilateral fEMG activation in each scene relative to baseline.

Scene	Muscle	Mean Diff	95% CI Low	95% CI High	t	*p*	dz	*p*(FDR)
Emotional	Frontalis	4.31	0.65	7.97	2.59	0.01	0.75	0.05 *
	Corrugator	3.66	−0.36	7.67	2.00	0.05	0.58	0.07
	Orbicularis	1.89	−2.80	5.68	0.89	0.19	0.26	0.26
	Zygomaticus	−0.06	−2.04	1.91	−0.07	0.52	−0.02	0.52
Cognitive	Zygomaticus	−2.15	−4.44	0.87	−1.66	0.13	−0.48	0.99
	Frontalis	−3.72	−9.71	2.27	−1.51	0.15	−0.43	0.99
	Orbicularis	−3.68	−6.53	−0.84	−2.85	0.01	−0.82	0.99
	Corrugator	−1.84	−4.73	1.05	−1.40	0.17	−0.4	0.998
Physical	Zygomaticus	3.56	0.22	6.89	2.32	0.04	0.67	0.05 *
	Orbicularis	4.82	0.42	9.23	2.19	0.04	0.63	0.04 *
	Frontalis	0.81	−5.93	7.55	0.27	0.39	0.08	0.53
	Corrugator	−0.36	−3.07	3.07	−0.30	0.38	−0.09	0.53
Dual	Zygomaticus	6.69	2.78	10.60	3.48	0.00	1.00	0.006 *
	Orbicularis	6.10	1.13	11.07	2.70	0.01	0.78	0.01 *
	Frontalis	−1.89	−8.37	4.58	−0.64	0.33	−0.19	0.33
	Corrugator	−1.47	−4.63	1.68	−1.02	0.83	−0.30	0.83

Note. Mean diff = mean difference between conditions; CI = confidence interval; t = t statistic; *p* = *p*-value; dz = Cohen’s d for paired samples; *p*(FDR) = *p*-value adjusted using false discovery rate correction. Asterisks (*) indicate statistically significant results after FDR correction (*p*(FDR) < 0.05).

Statistical non-significance, however, does not necessarily imply the absence of an effect; it may instead reflect insufficient evidence to detect a difference. Importantly, several muscle–scene contrasts did not differ significantly from baseline after FDR correction. While these results do not confirm equivalence to baseline, they are consistent with the absence of robust activation in non-target muscles, supporting the anatomical specificity of the fEMG signals and suggesting that observed activations reflect targeted muscle recruitment rather than generalized facial co-activation or baseline drift. To address this ambiguity, we complemented the superiority tests with a two one-sided test (TOST) equivalence procedure, using ±5%MVIC as the practical equivalence bound. This analysis evaluates a distinct hypothesis: whether muscle activation during a scene is meaningfully indistinguishable from baseline. By combining significance and equivalence testing, we distinguish three interpretable outcomes: (i) muscles showing reliable increases relative to baseline (superiority), (ii) muscles showing activation levels statistically equivalent to baseline (practical invariance), and (iii) results that remain inconclusive. This dual framework provides a more informative characterization of fEMG responses than reliance on significance testing alone.

Across all conditions, muscle activity remained low and bilaterally stable, confirming the reliability of the averaged signals. Scene-specific analyses nevertheless revealed clear, context-driven modulation of muscle activation. In the emotionally demanding scene (City), the frontalis showed a small but significant increase relative to baseline, suggesting heightened attentional engagement. The cognitive demanding scene (Mole) exhibited no significant activations, consistent with the baseline or resting facial state. In contrast, both the physical (Stroll) and dual (Stroll+CPT) scenes elicited strong increases in zygomaticus and orbicularis activation corresponding to positive-valence, smile-related expressions. These effects were moderate to large in magnitude, indicating robust emotional engagement.

### Appendix A.7. Detailed Mixed-Effects Model Results

This appendix provides full mixed-effects model results for all tested associations between facial expression features and NASA-TLX dimensions. All models include participants as a random effect and apply Benjamini–Hochberg FDR correction. Results are reported for transparency and completeness and should be interpreted as exploratory. For Emteq-derived dynamic features in the physical-demand scene (Stroll), NASA-TLX physical demand was negatively associated with several smile transition measures, including Smile→Smile count (β = −1.54, SE = 0.37, q = 0.0004), Neutral→Smile probability (β = −1.71, SE = 0.50, q = 0.0054), and Smile→Neutral count (β = −1.51, SE = 0.44, q = 0.0054). The corresponding Emteq-derived statistic features showed the same direction, including smile count (β = −1.54, SE = 0.37, q = 0.0005), average smile burst magnitude (β = −2.30, SE = 0.59, q = 0.0013), and smile burst count (β = −1.50, SE = 0.43, q = 0.0059). For our CNN–TCN–derived features, NASA-TLX physical demand in the cognitive-demand scene (Mole) was also negatively associated with smile persistence, including Smile→Smile count (β = −24.36, SE = 6.84, q = 0.0121), Smile→Smile probability (β = −1.86, SE = 0.54, q = 0.0155), and the statistic feature average smile burst magnitude (β = −5.01, SE = 1.42, q = 0.0329). Taken together, these convergent findings suggest that higher perceived physical workload is accompanied by reduced smile-related activity/persistence (broadly compatible with reduced FACS AU12-related activity), while remaining strictly correlational.

In the emotional-demand scene (City), Emteq-derived features additionally showed FDR-surviving covariations between workload dimensions and smile/eyebrow dynamics. NASA-TLX effort was positively associated with Smile→Smile count (β = 3.16, SE = 1.01, q = 0.0204), Smile → Neutral count (β = 1.82, SE = 0.59, q = 0.0209), and Neutral→Smile count (β = 1.87, SE = 0.62, q = 0.0220), alongside a negative association with Neutral→Neutral probability (β = −3.82, SE = 1.38, q = 0.0453). NASA-TLX frustration was positively associated with Neutral→Smile count (β = 1.45, SE = 0.48, q = 0.0294) and Smile→Neutral count (β = 1.39, SE = 0.46, q = 0.0294), while showing negative associations with eyebrow/surprise-related transitions including Neutral→Raised Eyebrow count (β = −2.49, SE = 0.86, q = 0.0357), and Raised Eyebrow→Neutral count (β = −2.46, SE = 0.86, q = 0.0357).

Finally, for our CNN–TCN–derived dynamic features in the physical-demand scene (Stroll), NASA-TLX performance was positively associated with Raised Eyebrow→Neutral probability (β = 1.51, SE = 0.47, q = 0.0066), suggesting that aspects of eyebrow-to-neutral recovery dynamics covary with perceived task performance in this context. Overall, under conservative multiple-comparisons control, robust effects concentrate primarily on smile-related dynamics (and more selectively on eyebrow-related transitions), indicating that workload-related facial expression modulation is detectable but modest and context-dependent. All reported associations reflect covariation rather than causal effects.
